# Should We Consider Neurodegeneration by Itself or in a Triangulation with Neuroinflammation and Demyelination? The Example of Multiple Sclerosis and Beyond

**DOI:** 10.3390/ijms252312637

**Published:** 2024-11-25

**Authors:** Océane Perdaens, Vincent van Pesch

**Affiliations:** 1 Neurochemistry Group, Institute of NeuroScience, Université Catholique de Louvain (UCLouvain), 1200 Brussels, Belgium; oceane.perdaens@uclouvain.be; 2 Department of Neurology, Cliniques Universitaires Saint-Luc, Université Catholique de Louvain (UCLouvain), 1200 Brussels, Belgium

**Keywords:** neurodegeneration, neuroinflammation, demyelination, multiple sclerosis, Alzheimer’s disease, Parkinson’s disease, metabolic syndrome

## Abstract

Neurodegeneration is preeminent in many neurological diseases, and still a major burden we fail to manage in patient’s care. Its pathogenesis is complicated, intricate, and far from being completely understood. Taking multiple sclerosis as an example, we propose that neurodegeneration is neither a cause nor a consequence by itself. Mitochondrial dysfunction, leading to energy deficiency and ion imbalance, plays a key role in neurodegeneration, and is partly caused by the oxidative stress generated by microglia and astrocytes. Nodal and paranodal disruption, with or without myelin alteration, is further involved. Myelin loss exposes the axons directly to the inflammatory and oxidative environment. Moreover, oligodendrocytes provide a singular metabolic and trophic support to axons, but do not emerge unscathed from the pathological events, by primary myelin defects and cell apoptosis or secondary to neuroinflammation or axonal damage. Hereby, trophic failure might be an overlooked contributor to neurodegeneration. Thus, a complex interplay between neuroinflammation, demyelination, and neurodegeneration, wherein each is primarily and secondarily involved, might offer a more comprehensive understanding of the pathogenesis and help establishing novel therapeutic strategies for many neurological diseases and beyond.

## 1. Introduction

Multiple sclerosis (MS) is a chronic immune-mediated, demyelinating, and neurodegenerative disorder of the central nervous system (CNS) affecting 2.8 million people worldwide [1,2]. Based on the criteria of disease activity and progression [3], it has been phenotypically divided into relapsing-remitting MS (RRMS), and progressive MS (PMS, primary (PPMS) when progressing from disease onset, secondary (SPMS) when following a relapsing-remitting course). RRMS patients present with subacute neurological deficits during relapses, which are the expression of an acute and focal inflammatory assault within the CNS, separated by longer periods of remission. In contrast, PMS patients experience ongoing disability worsening independently of relapses [3,4].

The pathogenesis of MS is still incompletely understood. Especially regarding its primary trigger, two dogmas are still in opposition. According to the outside-in theory, the infiltration of activated peripheral immune cells induces a central inflammatory, demyelinating, and neurodegenerating cascade. On the contrary, in the inside-out theory, a primarily unknown central insult releases autoantigenic myelin components, resulting in a secondary inflammatory (peripheral and central) response. Depending on the individual predisposition among other yet unknown factors, the immune response might be explosive (Marburg variant) to insidious (primary progressive MS) with a continuum in between (RRMS evolving into SPMS) [5]. Since women have a higher predisposition to autoimmunity, a 3:1 female-to-male ratio is observed in RRMS but not in PMS [6,7].

**Figure 1:**
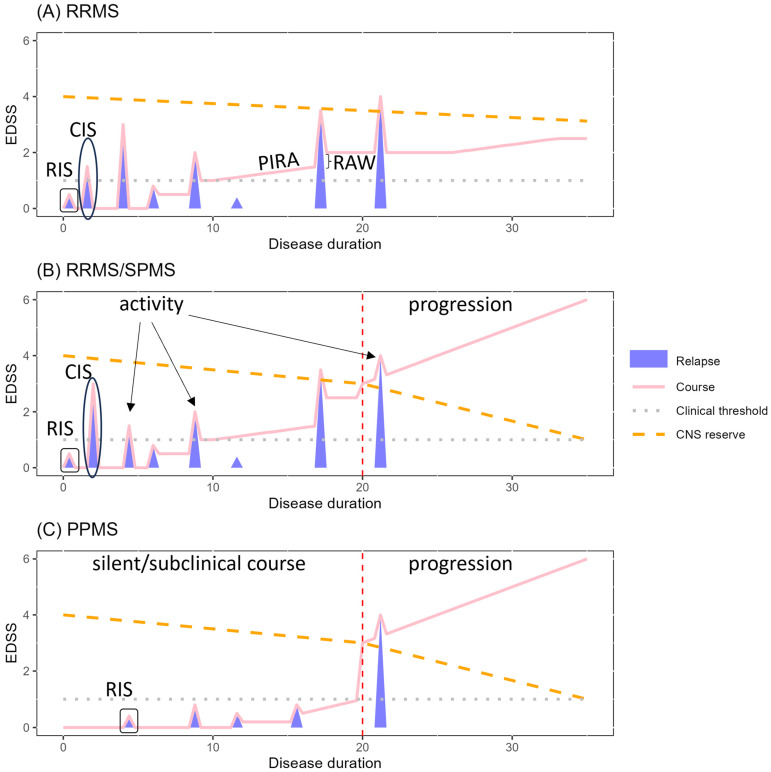
Disease course and phenotypes. In these plots, the disease course (pink solid line) and disability measured by the Expanded Disability Status Scale (EDSS) score (y-axis) are depicted according to disease duration (x-axis) for (**A**) relapsing-remitting multiple sclerosis (RRMS), (**B**) secondary progressive MS (SPMS), and (**C**) primary progressive MS (PPMS). Disease activity and its progression determine the disease course. The relapsing-remitting course, occurring in most patients, is characterized by acute inflammatory demyelinating events (blue pyramids) resulting either in (i) new lesions on MRI below the clinical threshold (grey dotted line) and/or (ii) clinical relapses with (partially) reversible disability worsening. These alternate with periods of remission. Early in the disease, patients may recover completely, but with increasing disease duration and aging, relapse-associated worsening (RAW) might occur. Disability progression independent of relapse activity (PIRA) also occurs, sometimes already early in the disease. In progressive MS, patients suffer from progressive worsening, not necessarily linked to the occurrence of inflammatory disease activity. Secondary progressive MS can occur after a relapsing/remitting course, while 10–15% of patients develop a progressive phenotype from disease onset, so-called primary progressive MS. The neuroaxonal reserve (orange dashed line) decreases over time, due to aging, neuroinflammatory, and demyelinating processes. Once the compensatory mechanisms become insufficient, disability progresses. A clinically isolated syndrome (CIS) corresponds to the first clinical demyelinating event. In a radiologically isolated syndrome (RIS), white matter lesions compatible with MS are discovered on MRI but any history of related neurological symptoms is missing.

Multiple sclerosis is heterogeneous in its clinical presentation (the distinctive phenotypes), but also by the ongoing and changing underlying pathophysiological mechanisms. This is further evidenced by the differences in inflammatory and demyelinating activity in the different types of MS lesions [8,9]. Active demyelinating plaques are associated with perivenous inflammation mediated by a bulk invasion of peripheral adaptive and innate immune cells through a disrupted blood–brain barrier (BBB) in acute and relapsing MS. On the contrary, a compartmentalized inflammatory response involving tissue-resident CD8+ T cells and B cells is associated with low-grade myelin and axonal loss at the margins of smoldering, slowly expanding white matter lesions containing macrophages/microglia and cortical subpial demyelination. These are more predictive of disease progression causing brain and spinal cord atrophy [2,10,11,12,13,14,15,16]. Recent knowledge has changed the paradigm of MS pathogenesis. Hereby, all phenotypes are now considered within a single continuum wherein inflammation and neurodegeneration coexist at varying levels during the disease course [17] (Figure 1). As SPMS can follow a relapsing-remitting course, PPMS might be preceded by a silent disease course in which acute inflammatory events remain subclinical [18]. Remarkably, PPMS and SPMS start approximately around the same age, on average in the fifth decade of life, yet in SPMS, progression is influenced by the relapse course within the first two years of disease onset but almost not by later relapses [6,19,20]. Therefore, progression becomes clinically evident when the compensatory mechanisms for axonal/neuronal loss and demyelination are exhausted, due to both disease mechanisms and senescence processes affecting all cell types and exacerbated by the disease pathogenesis. Within the immune system, these are referred to as inflammaging and immunosenescence [21,22].

Over the last two decades, treatment options have largely increased in MS. Current disease-modifying therapies (DMTs) mostly target the peripheral immune system and are efficient in reducing the relapse rate and thus controlling disease activity [23,24]. Of note, recent clinical trials evidenced disease progression independent of relapse activity (PIRA) in RRMS occurring already early in the disease, further highlighting the unconstrained burden of neurodegeneration [20]. The natural history of MS has remarkably shifted since the introduction of DMTs, mainly marked by a longer period between diagnosis of RRMS and onset of SPMS [25]. DMTs might thus have silenced the inflammatory processes, bringing the natural history of SPMS to that of PPMS, given that SPMS occurs at the same age as PPMS, nearly irrespective of the relapse history [5,6,19]. However, there is still no specific treatment for neurodegeneration-associated disease progression [23,26].

**Figure 2:**
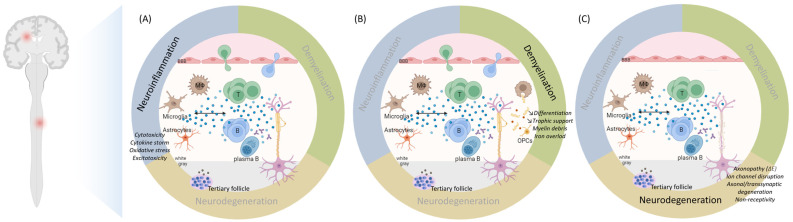
The pathogenesis of multiple sclerosis. (**A**) Neuroinflammation is marked by the invasion of peripheral immune cells in the CNS through a disrupted BBB in the early inflammatory phase of MS. These cells are reactivated, secrete cytokines (e.g., IFNg by Th1, IL6/17 by Th17, GM-CSF, IL6, TNFa by B cells) and cytotoxic molecules (e.g., granzyme B by CD8+ T cells), attract more peripheral immune cells and activate macrophages, microglia and astrocytes, which produce cytokines, nitric oxide, and reactive oxygen species (ROS) (blue dots). B cells can also differentiate into autoantibody-producing plasma cells. With disease progression, infiltration of peripheral immune cells is reduced since the BBB is closed. CNS-resident cells, i.e., microglia and astrocytes, sustain the inflammation by producing cytokines (TNFa, IL6) and releasing ROS (blue dots). TNFa-mediated glutamate release from microglia and its impaired turnover by astrocytes result in excitotoxicity. Remarkably, plasmablasts and plasma B cells form tertiary follicle-like structures in the meninges that may release proinflammatory factors activating microglia (brown dots). (**B**) Demyelination is partly caused by this cytotoxic and proinflammatory environment that breaks down the myelin sheaths. Macrophages and microglia, attracted by astrocytes, can clear the myelin debris, allowing (partial) remyelination by surviving oligodendrocytes or by OPCs proliferating, migrating, and differentiating at the site of injury in response to cytokines, chemokines (CXCL1, CXCL12), mitogens (platelet-derived growth factor), chemoattractants (semaphorin 3F), and trophic factors (insulin-like growth factor, ciliary neurotrophic factor) (blue dots) released by microglia and astrocytes. This will reduce the harm to the axons. However, the phagocytic capacity of microglia/macrophages decreases with disease progression. Hence, myelin debris are improperly cleared, trigger an inflammatory response, and inhibit axonal growth, and OPCs are less recruited and fail to differentiate. The trophic support of oligodendrocytes to the underlying axons wanes. The ferrous iron (red dots) released from the myelin, where it accumulates with age, is oxidized to ferric iron, producing ROS and causing lipid peroxidation and ferroptosis. (**C**) Neurodegeneration starts early in the disease and becomes prominent with disease progression when the compensatory mechanisms safeguarding the CNS reserve are exceeded. Axons are directly harmed by the proinflammatory and oxidative environment, but also by the loss of the insulating and supporting myelin sheaths. Chronically demyelinated axons seem to be non-receptive to OPCs. Nodal and paranodal ion channels are disorganized; synapses are dysfunctional. Axons suffer a major energy debt altering axonal transport of mitochondria and synaptic vesicles. The axonopathy spreads the axonal and transsynaptic degeneration. These events are self-sustained and intermingled, further enhanced by senescent processes, resulting in a major cytokine storm and oxidative burst, mitochondrial dysfunction, mitochondrial DNA damage, energy failure, ion imbalance, cytotoxicity, excitotoxicity, lack of trophic support by the loss of oligodendrocytes, and axonal loss. BBB = blood–brain barrier; B = B cell; CNS = central nervous system; ΔE = energy deficit: MS = multiple sclerosis; OPC = oligodendrocyte progenitor cell; ROS = reactive oxygen species; T = T cell; Th = T helper cell. Created in BioRender.com.

The pathogenesis of MS has long been fragmented in its understanding and research. This review presents a distinctive perspective, diverging from previous analyses, by examining the complex interactions between neuroinflammation, demyelination, and neurodegeneration. This approach offers an innovative framework for reevaluating multiple sclerosis and other neurodegenerative disorders, while simultaneously paving the way for novel therapeutic interventions in the future. We emphasize, through the example of MS, that neurodegeneration cannot be dissociated from neuroinflammation and demyelination. The definition of MS highlights the three preeminent facets of its unique pathogenesis (Figure 2). Herein, neuroinflammation, driven by both the innate and adaptive immune system, and demyelination overtly direct its pathogenesis, while neurodegeneration has long been underestimated. Moreover, the weight of each in the disease pathogenesis might determine the different phenotypes of MS. In particular, PMS reflects a diffuse functional and structural harm to the CNS rather than the sum of focally acquired demyelinating white and gray matter lesions. Each of these processes are also involved in other neurodegenerative disorders such as Alzheimer’s (AD) and Parkinson’s disease (PD), although in a different proportion [27,28,29,30,31,32,33,34,35]. Moreover, the metabolic syndrome causes a chronic low-grade systemic inflammation and has been linked to neurodegenerative disorders [36]. Thus, a better understanding of the complex intrication between these pathophysiological mechanisms could foster the search for novel and possibly complementary therapeutic strategies.

## 2. Neuroinflammation in MS

**Figure 3:**
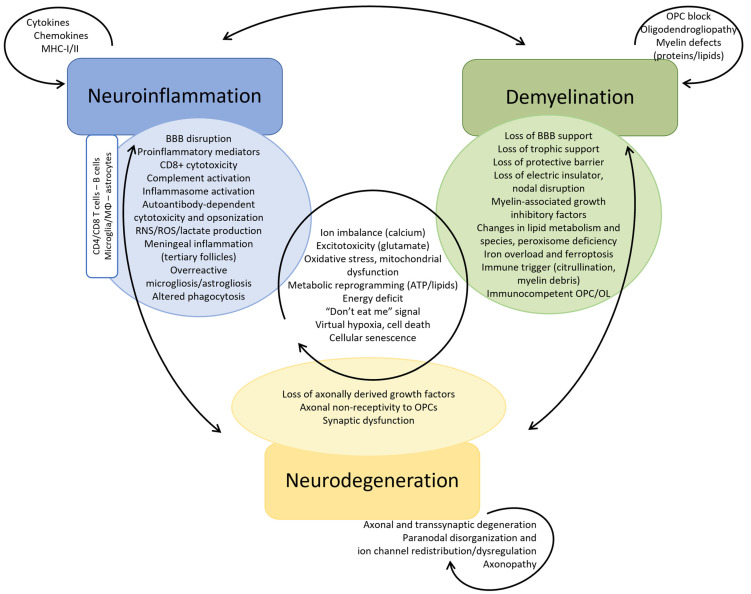
Triangulation of neuroinflammation, demyelination, and neurodegeneration in MS pathogenesis with key mechanisms that self-sustain these processes but also influence each other mutually. The close loops highlight underlying mechanisms that intrinsically sustain each process, i.e., neuroinflammation, demyelination, or neurodegeneration indicated in the rectangular boxes. The bubbles list pathophysiological mechanisms by which each process (with corresponding color) impacts at least one of the two other processes, while the bubble in the middle summarizes shared mechanisms occurring in all the cell types involved. BBB = blood–brain barrier, MS = multiple sclerosis, MΦ = macrophages, OPC = oligodendrocyte progenitor cell, OL = oligodendrocyte.

**Figure 4:**
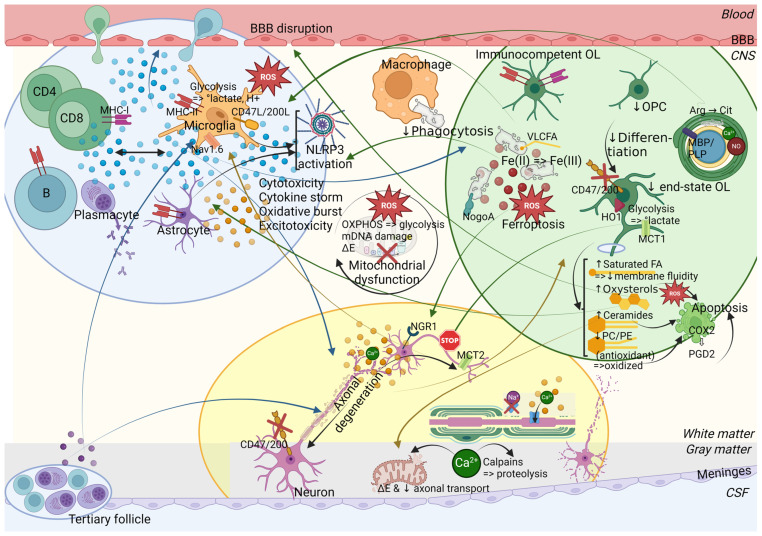
Pathophysiological mechanisms involved in the triangulation of neuroinflammation (blue bubble), demyelination (green bubble), and neurodegeneration (yellow bubble) in MS. The impact of each on the other processes is indicated by arrows in the corresponding color, whereas intrinsic effects are indicated by black arrows. *Neuroinflammation* (*blue bubble*): Peripheral immune cells and CNS-resident cells attract and activate each other through the release of cytokines and chemokines (blue dots), whereby MHC type I and II play an important role in antigen presentation. Cytokines also contribute to the disruption of the BBB. CD8+ T cells, reactive microglia, and astrocytes cause cytotoxicity. Plasma B cells release antibodies of partially unknown significance, which may contribute to the pathophysiology via antibody-dependent cellular toxicity, opsonization, complement-dependent cytotoxicity, and antibody-induced demyelination. The activation of microglia (which relies on glycolysis and lactate production with extracellular acidification) and astrocytes results in the production of ROS. Microgliosis and astrogliosis also involve NLRP3 inflammasome activation. The resulting proinflammatory and oxidative environment harms OPCs, oligodendrocytes, and neurons. High levels of glutamate (orange dots) (due to TNFa/ATP-induced release from microglia and reduced turnover by astrocytes) cause axonal/neuronal and oligodendroglial damage through excitotoxicity via NMDA receptors. Meningeal inflammation mainly consists of plasmablasts and plasma B cells, which may form tertiary follicle-like structures. It is correlated with subpial demyelination and CSF protein levels of TNF, IFNg, and CXCL13. Thus, they affect neurons directly or by releasing an unknown soluble factor (purple dots) that activates microglia. The CD47 and CD200 expressed on oligodendrocytes and neurons inhibit their phagocytosis by microglia by binding to CD47L and CD200L (“Don’t eat me” signal) but the former are downregulated. *Demyelination* (*green bubble*): Subclusters of OPCs and end-state oligodendrocytes are reduced, as well as the recruitment, proliferation, and differentiation of OPCs into oligodendrocytes. Myelin defects appear to begin at the inner layer and are caused by (i) the citrullination of MBP (i.e., the conversion of positively charged arginine (Arg) into uncharged citrulline (Cit)) altering the interaction of MBP with the plasma membrane, (ii) MBP phase transition resulting from increased calcium levels and causing myelin vesiculation, and (iii) protein S-nitrosylation, especially of PLP, caused by nitric oxide (NO). Moreover, the lipid species in the plasma membrane are altered, as evidenced by an increase in saturated fatty acids, which reduces membrane fluidity, an increase in oxysterols and ceramides, and a decrease in phosphatidylcholine (PC) and phosphatidylethanolamine (PE), which have antioxidant properties. Oxysterols (by inducing oxidative stress), ceramides and the oxidized forms of PC and PE cause oligodendrocyte apoptosis, which results in the expression of COX2 that mediates the production of proinflammatory prostaglandins (e.g., PGD2), thereby sustaining apoptosis. Oligodendrocyte apoptosis further induces gliosis, saturated fatty acids can promote microglial activation, while ceramides enhance Th1 cytokine production and cause mitochondrial dysfunction in neurons. Moreover, citrullinated MBP is an immunogenic trigger, and immunocompetent oligodendrocytes express MHC-I/II and interferon-responsive genes. OPC support to the BBB is decreased, and BBB disruption is further enhanced by oxysterols. Myelin debris are initially cleared by macrophages/microglia; however, their phagocytic capacity decreases with disease progression and cellular senescence. Uncleared myelin debris induce NLRP3 inflammasome activation in microglia. They halt OPC differentiation and expose myelin-associated inhibitory factors, such as reticulon 4, previously known as neurite outgrowth inhibitory factor (NogoA), as well as myelin-associated glycoprotein (MAG) and oligodendrocyte myelin glycoprotein (OMG), which bind to the Nogo receptor 1 (NGR1) expressed on axons, thereby inhibiting axonal growth and regeneration. They are also enriched in very long-chain fatty acids (VLCFA) by impaired beta oxidation in peroxisomes, which contributes to neurotoxicity and neuroinflammation. Myelin stores iron, which increases with age, but causes ferroptosis when the storage capacity of ferritin is exceeded. The released ferrous iron (Fe(II)) is oxidized to ferric iron (Fe(III)), resulting in the production of hydroxyl radicals. Microglial uptake of iron causes their dystrophy resulting in a second release of iron. Furthermore, oligodendrocytes express heme oxygenase 1 (HO1) via the nuclear factor erythroid 2-related (NRF2) pathway in response to oxidative stress, resulting in the production of ferrous iron (Fe(II)) and bilirubin, an antioxidant, in an attempt to counter it; however, this also forms an additional source of harmful ferrous iron. Finally, oligodendrocytes safeguard the trophic support of axons by the exchange of lactate (produced by glycolysis in oligodendrocytes) through monocarboxylate transporter 1 (MCT1, expressed in oligodendrocytes) and MCT2 (expressed in neurons); however, this lactate supply is impaired, resulting in an axonal energy deficit. *Neurodegeneration* (*yellow bubble*): Axonopathy results from mitochondrial dysfunction, ion imbalance (by nodal/paranodal disruption of ion channels linked to demyelination with loss of saltatory conduction), and energy deficit. Intracellular calcium overload, entering via the sodium/calcium exchanger in reverse mode, the mitochondrial calcium uniporter, and acid-sensing ion channels (activated by inflammation-linked tissue acidosis), and enhanced by glutamate, plays a key role in inducing calpains, which are proteases that degrade the cytoskeleton and reduce axonal transport. Moreover, mitochondrial permeability transition pores are formed when intracellular calcium levels increase and cause the leakage of mitochondrial solutes resulting in mitochondrial collapse. Axonopathy causes anterograde and retrograde axonal and transsynaptic degeneration. Cortical demyelination and neuronal apoptosis can result in brain atrophy. Chronically demyelinated neurons appear to be unreceptive to myelin expansions of differentiating oligodendrocytes and to express fewer growth factors supporting oligodendrocytes. Moreover, OPCs sense axonal synaptic dysfunction within neuron-to-OPC synapses. Finally, neurons can induce microglia by the release of ATP and ion imbalance (Nav1.6 expressed on microglia). *Mitochondrial dysfunction* is enhanced by oxidative stress (increased ROS/RNS, reduced antioxidant NRF2 pathway), which causes mitochondrial DNA (mDNA) damage (deletions), oxidizes lipids and proteins, and alters the mitochondrial respiratory chain (also due to mitochondrial DNA deletions). There is a metabolic shift from oxidative phosphorylation (OXPHOS) to glycolysis to allow a rapid ATP production despite its relative inefficiency. This results in virtual hypoxia and energy failure. Neurons (by reduced PPARGC1A expression, regulating mitochondrial function) and OPCs/oligodendrocytes (by their iron storage and reduced antioxidant mechanisms) are particularly vulnerable. BBB = blood–brain barrier, CNS = central nervous system, CSF = cerebrospinal fluid, ΔE = energy deficit, MHC-I/II = major histocompatibility complex type I or II, MS = multiple sclerosis, OPC = oligodendrocyte progenitor cell, OL = oligodendrocyte, RNS = reactive nitrogen species, ROS = reactive oxygen species, ↑ = increased, ↓ = decreased. Created in BioRender.com.

While it is still debated whether the primum movens of MS is peripheral (activation of the immune system against myelin epitopes) or central (CNS damage, demyelination, and microglial activation prior to the breakdown of the BBB and the invasion by peripheral immune cells), it is now accepted that, in addition to CD4+ T helper (Th) cells (Th1/Th17), cytotoxic CD8+ T cells, B cells as well as CNS-resident cells, namely microglia and astrocytes, play an important role in the disease [37,38,39,40,41]. Nevertheless, inflammation, especially driven by microglial activation, leads to mitochondrial dysfunction, energy failure and oxidative damage in the different involved cell types, amplified by aging processes and long-lasting accumulation of CNS damage [27,28] (Figure 3 and Figure 4, Supplementary Table S1).

### 2.1. Self-Sustained

The acute phase of the disease is characterized by the multifocal, recurrent invasion of the CNS by peripheral encephalitogenic, autoreactive CD4+ T cells, through an abnormally permeable BBB [2]. These T cells are reactivated in the CNS and release proinflammatory cytokines (e.g., interferon gamma [IFNg], interleukin [IL] 12, IL17, granulocyte-macrophage colony stimulating factor [GM-CSF]) and chemokines that attract more immune cells from the periphery (CD4+ and CD8+ T cells, B cells and plasma cells, monocytes and macrophages), and activate CNS-resident cells (microglia and astrocytes), while regulatory T cells are impaired in their suppressive function. This results in the distinctive lesions of the white matter characterized by focal perivenular inflammation, neuroaxonal damage, and gliosis, although cortical demyelination may occur early as well [2,12,38,39,42,43,44,45]. While the CD4+ T cells initiate the autoimmune processes but are less involved in later stages [46], cytotoxic CD8+ T cells become rapidly more abundant [38,39]. B cells release autoantibodies with an uncertain pathogenicity in MS, that however may potentiate the activation of autoreactive T cells and microglia, i.e., by opsonizing an endogenous (myelin) antigen [47,48,49,50]. B cells also secrete pro- (IL6, IL12, tumor necrosis factor alpha [TNFa], IFNg, GM-CSF, lymphotoxin) and anti-inflammatory (IL10 by regulatory B cells) cytokines and act as antigen-presenting cells, hereby supporting CD4+ and CD8+ T cells as well as myeloid cells [51,52,53,54,55,56,57,58]. Activated microglia and astrocytes release proinflammatory cytokines and chemokines. Thereby, they promote their own activation via a direct autocrine (e.g., microglial TNFa) or paracrine (e.g., microglial IL1b on astrocytes, astrocytic IL6, and lymphotoxin-alpha on microglia) positive feedback loop. They recruit and reactivate peripheral immune T and B cells within the lesions, that clonally expand and further activate microglia and astrocytes. They also release anti-inflammatory cytokines (e.g., microglial IL10-inducing astrocytic transforming growth factor beta [TGFb] which in turn attenuates microglial activation) to restrain mutual inflammatory processes [28,40,59,60,61,62,63,64,65,66,67]. Reactive astrocytes may release both beneficial (retinoic acid, peroxiredoxin 6, sonic hedgehog) and detrimental factors (cytokines, chemokines, matrix metalloproteinases [MMPs], and reactive oxygen/nitrogen species [ROS/RNS]) affecting BBB integrity [66,68,69,70]. However, the ablation of reactive astrocytes increased the leukocytic CNS-infiltration, resulting in a fulminant course of experimental autoimmune encephalomyelitis (EAE) [71]. Astrocytes are thus required to contain the inflammatory assault [72]. Herein, they induce the expression of ectoendonucleases CD39 and CD73 in activated CD4+ T cells and hereby partially reverse the balance toward an immunoregulatory response [73]. During the reparative process, proliferative astrocytes form a glial scar around the lesion to segregate necrotic nerve tissue and inflammatory cells from the surrounding healthy tissue [74].

The chronic phase of the disease is characterized by a diffuse and compartmentalized inflammation, behind a functional and closed BBB. T cells, in particular CD8+ tissue-resident memory cells, and B cells relocate to the perivascular Virchow–Robin and meningeal spaces [2,16,75,76]. Hereby, B cells take a more prominent role and can form tertiary follicle-like structures that are associated with an earlier onset of PMS [77,78]. Moreover, more pronounced meningeal inflammation was associated with increased CSF-levels of proinflammatory cytokines and chemokines, in particular TNF, IFNg, and CXCL13, as well as molecules involved in B cell recruitment, function, and development [79]. Both microglia and astrocytes can exhibit a wide spectrum ranging from a pro- to an anti-inflammatory phenotype. Although they are able to contain the damage in the acute phase, overreactive microglia and astrocytes, sustained by inflammation-induced mitochondrial dysfunction, interact with other immune cells and glial cells via the release of proinflammatory cytokines and glutamate, the activation of the complement system as well as the production of ROS/RNS [80,81,82,83,84,85,86,87]. Moreover, inflammasome activation is involved in microgliosis and astrogliosis [88]. Microglial nucleotide-binding oligomerization domain, leucine-rich repeat, and pyrin domain-containing 3 (NLRP3) inflammasome induces neurotoxic astrocytes and contributes to cognitive impairment in the late phase of EAE [89]. Microglia and astrocytes thus support a self-sustained chronic inflammation-promoting disease progression.

### 2.2. Impacting Demyelination and Neurodegeneration

Proinflammatory cytokines released by peripheral and CNS-resident immune cells may harm myelin, neurons, and glial cells, causing demyelination, axonal transection (even in acute lesions), and cell death, which alters axonal conduction and exposes the axons to the proinflammatory environment, further contributing to axonal degeneration [90,91,92]. Both CD4+ and CD8+ T cells can induce microtubule axonal destabilization via lytic granules [93]. Th17 cells can form immune synapses with oligodendrocyte progenitor cells (OPCs) and axons and induce a partially reversible intra-axonal calcium influx, but therapies targeting solely CD4+ T cells (e.g., via a monoclonal antibody against the p40 subunit of IL12 and IL23) are inefficient in MS [94,95,96]. Cytotoxic CD8+ T cells target axons and oligodendrocytes through antigen presentation by major histocompatibility complex class I (MHC-I) molecules and granule exocytosis (containing perforin, granzyme B) in immune synapses, directly causing oligodendrogliopathy, demyelination, axonal damage, and neurotoxicity/neuronal apoptosis [97,98,99,100,101,102,103,104]. Their interaction with CD4+ T cells seems indispensable [46,105]. Noticeably, the CSF of MS patients contains elevated, neurotoxic levels of granzyme B and IL1B and the expression of granzyme B in peripheral CD8+ T cells of SPMS patients positively correlates with the progression of clinical symptoms [106,107].

The high prevalence of oligoclonal bands (OCBs) in MS patients as well as the efficacy of B cell depletion therapies support the involvement of B cells, in particular clonally expanded plasmablasts, but also memory B cells, and plasma cells [108,109,110,111,112,113,114]. OCBs remain stable over the years in MS patients. Their absence predicts a more benign course (possibly because of reduced plasma cell invasion), while the presence of IgM OCBs was associated with MS conversion (from CIS to RRMS and progression to SPMS), an increased relapse rate, and disability score [78,115,116,117,118,119,120]. The specific targets of OCBs are still unknown. The presence of autoantibodies against epitopes of myelin proteins such as myelin basic protein (MBP), proteolipid protein (PLP), or myelin oligodendrocyte glycoprotein (MOG), remains controversial [52,121,122,123,124,125]. Antibodies against Epstein–Barr virus (EBV) proteins have been identified as well, whereby molecular mimicry was evidenced between the EBV nuclear antigen 1 (EBNA1, an EBV transcription factor) and glial cell adhesion molecule (GlialCAM) [126,127,128]. However, antibodies were found in areas with myelin breakdown and have been implicated in demyelination by several mechanisms: (i) antibody-dependent cellular cytotoxicity by release of inflammatory components by innate immune effector cells (e.g., macrophages or granulocytes) expressing the Fc-gamma receptor, that recognizes and binds the gamma chain of the antigen-bound antibody complex, (ii) cell-induced demyelination via opsonization and phagocytosis of antibody-bound antigens (e.g., myelin) by phagocytotic cells expressing the Fc-gamma receptor, (iii) complement-dependent cytotoxicity and demyelination by antibody-dependent activation of the complement cascade and assembly and deposition of the membrane attack complex at sites of active myelin destruction, and (iv) direct antibody-induced demyelination, wherein the crosslinking of anti-MOG antibodies with MOG complexes resulted in the phosphorylation of specific proteins related to cellular stress response and cytoskeletal stability leading to retraction of oligodendrocyte processes [53,54,129,130,131,132,133,134,135,136,137,138,139,140,141]. Antibodies directed against the axo-nodal protein neurofascin, detected in the serum of MS patients, exacerbated the clinical course of MOG-induced EAE by causing axonal injury without demyelination nor enhanced CNS-inflammation. These antibodies inhibited axonal conduction in a complement-dependent manner [142]. Furthermore, B cells of RRMS patients have a direct or indirect (through activation of microglia or astrocytes) cytotoxic effect on oligodendrocytes and neurons that is independent of immunoglobulins by secreting (a) currently unidentified soluble toxic factor(s) [82,143,144]. Thereby, gray matter demyelination, widespread neuronal loss in subpial cortical lesions, and cortical atrophy corroborate with the extent of meningeal inflammation, alongside CSF protein levels of TNF, IFNg, and CXCL13, and is even more pronounced in the presence of tertiary follicle-like structures [77,79,91,144,145,146,147]. This is associated with higher and earlier disability [79]. Persistent intrathecal expression of TNFa and IFNg could induce meningeal inflammation in vivo, causing subpial demyelination and neuronal death by necroptosis [148]. Noticeably, a subtype of excitatory projection neurons was selectively vulnerable and reduced in demyelinated areas within the upper cortical layers, commonly underlying meningeal inflammation marked by infiltrating plasma cells. Single-nucleus RNA sequencing of these neurons outlined the upregulation of oxidative stress, mitochondrial dysfunction, and cell stress and cell death pathways [149].

Activated microglia appear in the periphery of chronic active, slowly expanding lesions, often loaded with iron, congregating low-grade, smoldering inflammation. These lesions are associated with chronic axonal damage and concurrent low-grade demyelination and are thus predictive of progression in RRMS and SPMS [12,15,150,151,152]. Activated microglia also spread diffusely throughout the brain and are strongly involved in diffuse axonal and neuronal damage in the normal appearing white (NAWM) and gray matter, as well as in cortical subpial and deep gray matter demyelination [10,144,153,154,155,156,157,158]. In fact, neurodegeneration is more closely linked to diffuse injury in the NAWM than the white matter lesion load/demyelination extent [10,159]. Although the NAWM appears macroscopically normal, it shows microscopically normally myelinated axon fibers but reduced axonal densities [160,161]. The diffuse pathology within the normal appearing white and gray matter starts early in disease but expands with disease progression [162,163]. It is partially linked to Wallerian degeneration following axonal transection in white matter lesions but is rather closely associated with diffusely scattered CNS-inflammation and cortical lesion volume and is thus partially independent of focal demyelination [10,21,97,158,164]. Chronic inflammation further induces glutamate release by glial cells causing nodal and paranodal disruption in the NAMW [165]. Remarkably, the extent of paranodal axoglial disruption is correlated with local microglial inflammation and axonal injury in NAWM, but not with demyelinating lesions and infiltrating lymphocytes [166].

Gene expression studies evidenced that microglial activation, oxidative burst, and DNA damage are more pronounced in areas of cortical lesions, associated with oligodendrocyte and neuronal injury [154]. Moreover, complement C3 upregulation by activated microglia mediates hippocampal dendritic loss and memory impairment in early stage EAE [167]. Microglia can induce, via TNFa and the complement C1q, neurotoxic astrocytes, that impair neuronal outgrowth and OPC migration, maturation, and differentiation, by expressing certain molecules (such as Netrin1, Jagged) and by releasing several other molecules (such as hyaluronan, fibronectin, chondroitin sulfate proteoglycans, fibroblast growth factor) in the extracellular matrix [65,166,168,169,170,171,172]. Reactive astrocytes can also promote TNF-mediated OPC cell death [173]. Activation of inflammasome complex component NLRP3 mediates via downstream caspase-1 and IL18 microglial activation and astrogliosis accompanied by enhanced demyelination and oligodendrocyte loss [174]. Furthermore, the neuron- and oligodendrocyte-anchored immunoregulatory “Don’t eat me”-signals, CD200 and CD47, dampen the activity of their receptor/ligand on microglia and macrophages, but in chronic active and inactive MS lesions, CD200 and CD47 expression is reduced, which is accompanied by increased microglial activation, complement expression at the lesion rim, and axonal and oligodendroglial damage [175,176,177,178,179].

Microglial activation is supported by a metabolic shift from oxidative phosphorylation (OXPHOS), predominant in homeostatic microglia, to glycolysis in activated microglia, to allow rapid ATP production despite its relative inefficiency, in order to rapidly support the production of proinflammatory cytokines in microglia, but this alters phagocytosis [180,181,182]. Moreover, with excessive aerobic glycolysis, increased lactate production contributes to extracellular acidification. Metabolic reprogramming in microglia can thereby enhance neuroinflammatory processes and neuronal damage [180,183,184,185].

Inflammation and oxidative stress are closely linked as they are mutually causative and sustainable [186]. Herein, microglia, macrophages, and astrocytes play a preeminent role by producing ROS and RNS (via NADPH oxidase, myeloperoxidase) [187,188]. ROS/RNS can damage mitochondrial and cellular components, oxidize mitochondrial DNA (lacking protective histones), lipids and/or membrane proteins. ROS/RNS inhibit the mitochondrial respiratory chain and mitochondrial DNA damage itself affects the transcription of its subunits, which further compromises OXPHOS and results in liberation of electrons nourishing ROS, thus amplifying the oxidative stress. Proinflammatory cytokines can also alter mitochondrial components and the enzymes of the tricarboxylic acid (TCA, or Krebs) cycle and of OXPHOS [27,81,154,189,190]. Moreover, mitochondrial dysfunction contributes to virtual hypoxia (reduced oxygen consumption and energy failure in conditions of normal blood and oxygen supply) damageable to the tissue. This is enhanced by true hypoxia with reduced oxygen supply due to severe inflammation, especially in watershed areas (at boundaries between blood supply territories of several cerebral arteries) where the lesion load is increased [27,191,192,193,194].

Thus, the imbalance between the production of free radicals vs. antioxidant molecules, i.e., by the decline in the neuroprotective nuclear factor erythroid 2-related factor 2 (Nrf2) antioxidant pathway, generates oxidative stress, leading to mitochondrial dysfunction, energy deficits, and ion imbalance in neurons, oligodendrocytes, and OPCs, mirrored by the expression of proteins linked to hypoxia, cellular and endoplasmic reticulum stress, and by the translocation of apoptosis-inducing factors [195,196,197,198,199,200,201,202,203,204,205,206]. OPCs and oligodendrocytes are threatened by ROS/RNS due to their limited antioxidant defense mechanisms and high iron content. OPCs are subsequently unable to differentiate into myelinating oligodendrocytes [205,207,208,209]. Moreover, nitric oxide can alter myelin architecture and cause its decompaction by protein S-nitrosylation, especially of PLP [210]. In neurons, the reduced expression of the transcriptional co-activator, peroxisome proliferator-activated receptor gamma coactivator 1-alpha (*PPARGC1A*), regulating mitochondrial function, and increased levels of clonally expanded mitochondrial DNA deletions are associated with decreased expression of OXPHOS subunits and antioxidants, thereby supporting their vulnerability [211,212]. Axonal mitochondrial dysfunction was evidenced prior to the onset of neurological symptoms in EAE. Mitochondrial dysfunction was associated with reduced mitochondrial trafficking and correlated in number and location with infiltrating immune cells, especially NO-producing macrophages as well as activated microglia and astrocytes at the onset of neurological deficit in the absence of demyelination [213]. Axons are susceptible to mitochondrial dysfunction, enhanced by demyelination and subsequent exposure to the deleterious environmental conditions. As axonal transport is highly energy demanding, its early compromise accelerates axonal damage [214,215,216]. Furthermore, mutations in mitochondrial DNA and reduced expression of nuclear DNA-encoded mitochondrial proteins and axonal motor proteins cause axonal degeneration while they are better tolerated in small cells [211,216,217].

As a result, oxidative stress causes functional impairment without structural damage when it is mild, with structural damage when a certain threshold is reached, resulting in neuroaxonal damage and demyelination, and ultimately apoptotic cell death and tissue destruction [27,218,219,220]. In inactive plaques, axonal mitochondria are increased (in content, size, activity, speed of movement) to respond to the increased energy demand. In surviving chronically demyelinated axons, remyelination partially reduces mitochondrial numbers and function but these remained higher than in unaffected myelinated axons [221,222,223]. Brain autopsy samples of MS patients have a higher proportion of neurons with mitochondrial dysfunction than in controls and cortical neurons with mitochondrial DNA deletions are equally distributed through the cortex, independent of the presence of cortical lesions [211].

An astrocytic–microglial interplay also mediates excitotoxicity [224]. Excitotoxicity refers to the excessive stimulation of excitatory metabotropic glutamate receptors (mGluR1, mGluR5) and ionotropic receptors (alpha-amino-3-hydroxy-5-methyl-4-isoxazolepropionic acid (AMPA), kainate, N-methyl-D-aspartate (NMDA)), induced by glutamate. This causes axonal/neuronal and oligodendroglial damage and cell death by direct cytotoxicity or by increasing intracellular calcium concentrations (by influx or by mobilization of intracellular stocks), which induces lipid peroxidation via the formation of free radicals and ROS [225,226,227,228,229,230,231,232,233,234]. Glutamate release by activated microglia/macrophages and leukocytes is enhanced by the release of microglial ATP and proinflammatory cytokines such as TNFa and IL1b. Moreover, its uptake by astrocytes is impaired due to the loss of glutamate transporters, resulting in increased extracellular glutamate concentrations [224,235,236,237,238]. Remarkably TNFa-dependent excitotoxic cell death occurred in murine brain slices incubated with CSF of PMS patients [236]. Glutamate concentrations are increased in active lesions and the NAWM as well as in the CSF of MS patients during relapse and are associated with brain volume loss [239,240,241,242]. Glutamate ionotropic receptor NMDA type subunit 2A (GRIN2A), a subunit of the NMDA receptor, has been identified as a susceptibility gene for MS risk and severity [243,244]. Similarly, a higher genetic score, corresponding to the total number of risk alleles (although without GRIN2A) linked with higher baseline glutamate levels, correlated with higher gray matter glutamate concentrations and brain atrophy at 1-year follow-up [245]. Furthermore, tryptophan and quinolinic acid can also contribute to excitotoxicity. Tryptophan is mainly metabolized via the kynurenine pathway, leading to the production of kynurenic acid (neuroprotective) or quinolinic acid (neurotoxic). During acute neuroinflammation, the production of kynurenic acid is predominant, but this pathway is shifted during chronic enzymatic activation toward the production of neurotoxic metabolites. Quinolinic acid is also an agonist of NMDA receptors, inducing synaptic glutamate release, inhibiting its reuptake and reducing its conversion to glutamine [246,247].

However, microglia can also be neuroprotective by the transition from a pro- to an anti-inflammatory/proregenerative phenotype, presumably via necroptosis of the former [248,249]. In fact, two distinct microglia clusters have been identified in the chronic active lesion edge, i.e., an iron cluster characterized by MHC-II and inflammatory markers (especially IL1b and complement C1q) as well as iron-related genes, thus involved in microglial activation and reactive microgliosis, and a foamy cluster characterized by foam-cell differentiation and lipid storage, thus involved in myelin phagocytosis [85]. They are recruited by astrocytes and involved in the clearance of myelin debris, which is indispensable for OPC differentiation and remyelination [250,251,252,253,254]. Microglia and astrocytes promote the recruitment, proliferation, and differentiation of OPCs as well as axonal regeneration and neurogenesis, i.e., by secreting several chemotactic molecules, neurotrophic factors, and growth factors (insulin like growth factor 1 [Igf1], activin A) [171,252,255,256,257,258,259,260]. Thereby, incomplete demyelination in periplaque regions suggests an attempt of tissue remodeling in PMS spinal lesions, alongside a reduced axonal density. However, despite the superimposition of a pro- and anti-inflammatory transcriptomic signature, the poor phagocytic activity of macrophages/microglia, the altered function of astrocytes, and low-grade inflammatory events prevail in these lesions [261]. Hence, the protective/reparative processes fail with disease progression due to the sustained proinflammatory activation as seen by the preferential accumulation of proinflammatory microglia-expressing genes involved in immune defense and inflammatory processes at the edge of slowly expanding lesions [87,262].

In conclusion, the interplay between microglia and astrocytes is the driver of self-perpetuating focal and diffuse neuroinflammation and modulates the oxidative balance and synaptic conduction. Their chronic (over-)activation leads to oxidative stress, excitotoxicity, and an anti-regenerative microenvironment, causing neurodegeneration, demyelination, and failure of remyelination and self-repair in the progressive phase of MS [28,152].

## 3. Demyelination in MS

Myelin is naturally degraded, and its turnover is ensured by the pool of OPCs in the adult brain (accounting for 5–10% of cells within the CNS) [263]. ROS cause damage to myelin sheaths and facilitate macrophage/microglia activation. OPCs and oligodendrocytes are harmed by the inflammatory environment, the oxidative stress and the direct cytotoxicity generated by CD8+ T cells, reactive microglia and astrocytes [90,143,264,265,266,267,268,269]. OPCs and oligodendrocytes are sensitive to oxidative stress due to their limited antioxidant capacity and high iron content, which hampers their differentiation to oligodendrocytes and prompts cell death [207,270,271,272]. On the contrary, the inside-out theory postulates that a primary cytopathy affecting oligodendrocytes and myelin by a yet unknown mechanism might be the earliest event, possibly years before the first symptoms. Hereby, the shedding of myelin debris will trigger a secondary immune response marked by T and B cell infiltration depending on the host’s predisposition to respond to these autoantigenic components [5,273,274].

Remyelination occurs at the beginning of the disease, thanks to surviving oligodendrocytes and to the proliferation, migration, and differentiation of OPCs upon signals emanating from microglia/astrocytes and clearance of myelin debris [252,258,259,275,276,277,278,279]. There seems to be a critical window of time promiscuous for remyelination, beyond which demyelination-related axonal damage and thus functional loss cannot recover completely [280]. However, newly formed myelin is thinner and shorter [281]. Its efficiency declines with age and this is further accelerated by disease progression due to impaired OPC recruitment and differentiation [282,283,284]. Moreover, remyelination is often incomplete and limited to the lesion borders as only approximately 20% of chronic lesions are completely remyelinated, alongside hypomyelinated shadow plaques [285]. Incompletely myelinated axons with shorter internodes are more susceptible to neurodegeneration due to the inappropriate redistribution of juxtanodal components and nodal sodium channels [281,286] (Figure 3 and Figure 4, Supplementary Table S1).

### 3.1. Self-Sustained

Single-cell RNA sequencing identified similar OPC and oligodendrocyte subclusters in MS patients and controls, although in a different proportion. It also highlighted changes in their transcriptional signatures and an alteration in oligodendroglial heterogeneity in MS lesions and the NAWM. Several oligodendrocyte subclusters were enriched in MS, namely a mature actively myelinating subcluster, an end-state and an immune oligodendrocyte subcluster, the latter expressing immune genes (such as CD74 (a component of MHC-II), complement C3 and C1QB) and being closely associated with microglia. Oppositely, OPC and intermediate oligodendrocyte subclusters were underrepresented. Notably, the reduction in another end-state oligodendrocyte subcluster that was transcriptionally directed toward signaling, cell-to-cell adhesion, and viability rather than myelination might be important in the understanding of MS pathogenesis [287].

Citrullination accompanies ongoing demyelination in active and chronic active lesions, where myelin swelling and MBP citrullination and degradation are increased [288]. Myelin-associated peptidyl arginine deiminase (PAD) is upregulated in spontaneously demyelinating transgenic mice prior to the onset of clinical or pathological signs of demyelination [289]. Moreover, myelin of MS patients is developmentally immature as it is enriched in citrullinated MBP, as seen in early childhood, as well as PAD [290,291]. However, it is still debated whether PAD is enhanced in the NAWM [290,292]. This calcium-dependent enzyme is responsible for the conversion of positively charged arginine to uncharged citrulline, thereby causing a primary defect in the interaction of MBP with the plasma membrane and with other molecules [293]. Moreover, elevation of intracellular calcium levels resulted in MBP phase transition and network disassembly causing myelin vesiculation at the inner layers [294]. Myelin defects may thus begin at the inner myelin sheath while the outer layers remain intact and even occur beyond areas of inflammation.

Likewise, in a subset of active lesions, a primary oligodendrogliopathy is characterized by the early degeneration of distal, periaxonal oligodendrocyte processes, and by the loss of myelin-associated glycoprotein (MAG) expression but a prominent nuclear expression of hypoxia inducible factor 1a (HIF1a) [295,296]. Oligodendrocyte apoptosis resulted in rapid demyelination. It is accompanied by a localized gliosis and microglial activation, in the absence of peripheral immune cell infiltration [297,298,299,300]. Macrophages may then be recruited for the clearance of myelin debris, while T and B cells were only evidenced in recently demyelinated tissue, with possibly already signs of oligodendrocyte regeneration [300,301]. Early loss of oligodendrocytes is prominent in tissue bordering rapidly expanding MS lesions [301].

In MS, lipid and fatty acid metabolism is altered, resulting in the reduction in circulating polyunsaturated fatty acids, prone for (per)oxidation, alongside the compensatory increase in saturated fatty acids with a shorter carbon chain, that reduce the membrane fluidity [302]. Myelin is highly enriched in lipids, with a unique composition (cholesterol/phospholipids/glycolipids in a 2:2:1 ratio), whereby lipids form microdomains, called lipid rafts, that are important for the guidance of membrane proteins, trafficking, and signaling [303]. Lipid and energy metabolism as well as myelin turnover by macroautophagy and lysosome-mediated degradation of lipids into fatty acids are prominent in the physiology and pathophysiology of OPCs/oligodendrocytes [304]. However, they decline with aging and pathological conditions, thereby affecting the well-being of oligodendrocytes, the stability and structural integrity of the myelin sheaths, and their ability to interact with myelin proteins [304,305,306]. Moreover, different serum metabolomic/lipidomic signatures were associated with MS (decrease in two phospholipids, namely phosphatidylcholine and phosphatidylethanolamine, with antioxidant properties) and disease severity (increase in lysophospholipids and oxidized fatty acids), which may reflect the activation of the immune system (lipids and amino acids as signaling molecules) or changes in CNS lipid composition due to myelin destruction [307,308]. Subtle changes in myelin lipid biochemical signatures were detected even in the NAWM [309]. Oppositely, oxidized phosphatidylcholine is harmful for oligodendrocytes and neurons [310]. Sphingolipid biosynthesis, de novo or by degradation of sphingomyelin, is enhanced by the proinflammatory impulses and/or oxidative stress, resulting in the generation of ceramide species that are interconvertible into sphingosine, both inducing oligodendroglial apoptosis [311,312,313,314,315]. Increased sphingosine levels in MS brains may thus contribute to demyelination [311]. Oxysterols can also cause oligodendrocyte cell death by inducing simultaneously oxidative stress, apoptosis, and autophagy [316]. Finally, apoptotic oligodendrocytes express cyclooxygenase 2 (COX2) in Theiler’s murine encephalomyelitis virus-induced demyelinating disease [317,318]. COX2 mediates the metabolism of arachidonic acid into active lipid mediators, i.e., prostanoids, among which proinflammatory prostaglandins PGD2 and d15-PGJ2 induce apoptosis of OPCs [319].

OPCs and oligodendrocytes rely on glycolysis for ATP production. Under stress conditions, oligodendrocytes can withdraw their processes to reduce their metabolic needs, adopting a survival modus, while OPCs cannot and also rely on a higher rate of oxidative metabolism, rendering them more vulnerable to cell stressors and subsequent cell death as seen in MS [275,320]. Moreover, hypoglycemic conditions reduce the proliferating, differentiating, and myelinating capacity of OPCs and oligodendrocytes. This can be rescued by lactate uptake via monocarboxylate transporter 1 (MCT1), which is, however, downregulated in OPCs, but not in oligodendrocytes, under prolonged deprivation conditions [275,321,322,323]. CSF lactate levels of MS patients increase in the acute phase and even more as disease progresses, reflecting an increased extra-mitochondrial glucose metabolism due to mitochondrial dysfunction [324].

### 3.2. Impacting Neuroinflammation and Neurodegeneration

Oligodendrocytes safeguard axonal integrity by two means. First, myelin ensures the insulation of axons allowing a time- and energy-saving electrical saltatory conduction. This relies on the highly organized assembly of Ranvier nodes, enriched in voltage-gated sodium channels, alongside paranodal axo-glial junctions where several adhesion molecules anchor the myelin loops to the axon [325]. Demyelination causes a diffuse redistribution of the nodal/paranodal/juxtaparanodal ion channels and molecules, whereas remyelination requires the aggregation of these molecules to restore nerve conduction and possibly prevent or lessen secondary axonal degeneration [286,325,326]. Secondly, an axon–oligodendrocyte interaction fine-tunes, independently of the myelin sheath, axonal energy demands during neuronal conduction, as the synaptic release of glutamate during action potential propagation induces NMDA receptors in oligodendrocytes that translocate glucose transporter 1 (GLUT1) into their membrane to fuel glycolysis [327]. Oligodendrocytes can then ensure trophic and metabolic support to axons by shuttling pyruvate/lactate via MCT1 (expressed in oligodendrocytes)—MCT2 (expressed in neurons) which allows to generate mitochondrial ATP and thus ensures mitochondrial function and transport within axons [328,329,330,331]. Reduced mitochondrial complex IV activity in demyelinated axons enhances glutamate-mediated axon injury [221]. Demyelination and insufficient remyelination exposes axons to extracellular stressors and thus to oxidative stress and increases energy demands by the ectopic redistribution of sodium and calcium channels along the denuded axolemma, that are normally only localized at the Ranvier nodes and the nerve terminal, respectively, together with the impaired lactate supply to axons, resulting in an energy deficit [33,286,330,332,333]. Moreover, ion channels fail to maintain the sodium/potassium flux needed for the propagation of the action potential [334,335]. Hence, primary oligodendrocyte death leads to inflammation-independent axonal damage with subcellular changes and loss of symbiotic interactions, even before demyelination occurs [336,337]. This is further supported by a mouse model in which toxin-induced focal subpial demyelination results in the selective degeneration and functional impairment of an interneuron subtype (characterized by the staining of calcium-binding protein parvalbumin), that is normally myelinated and particularly ATP-demanding and thus depending on nutrient supply by oligodendrocytes. The selective loss of these inhibitory interneurons has also been observed in MS brains [299]. Demyelination further facilitates transsynaptic degeneration [338].

Chronic demyelination causes neuronal apoptosis by inducing the mitogen-activated protein kinase (MAPK) stress pathway via dual leucine zipper kinase and by phosphorylating downstream c-Jun. Neuronal apoptosis can experimentally be prevented by remyelination [339]. Myelin forms an oxidative buffer for axons, as oxidative stress induces the expression of heme oxygenase 1 (HO1) via Nrf2 in oligodendrocytes, resulting in the production of ferrous iron (II/Fe^2+^) and bilirubin, an antioxidant. However, in a chronic setting, this additional source of ferrous iron is harmful by contributing to iron overload [199,340,341]. Hence, the impact of HO1 overexpression in EAE is still disputed [342,343]. Moreover, uncleared myelin debris inhibit OPC differentiation [251,344]. They also expose myelin-associated inhibitory factors, such as reticulon 4, previously known as neurite outgrowth inhibitory factor (NogoA), as well as MAG and oligodendrocyte-myelin glycoprotein (OMG), that bind to Nogo receptor 1 expressed on axons, and thereby inhibit axonal growth and regeneration [345,346,347]. Finally, myelin debris clearance induces a proinflammatory phenotype in foamy macrophages/microglia that negatively impact the disease by releasing inflammatory and toxic mediators and by presenting brain-derived autoantigens. In a second phase, they adopt an anti-inflammatory phenotype upon activation of the nuclear liver X receptor (LXR) and peroxisome proliferator-activated receptor (PPAR) by myelin-processed lipid metabolites. However, aging reduces their ability to process the cholesterol-rich myelin debris, resulting in the formation of cholesterol crystals and the activation of the NLRP3 inflammasome [348].

Oligodendrocyte metabolic dysfunction can impact energy supply to neurons and contribute to neurodegeneration [330,349]. Beta oxidation in mitochondria and peroxisomes is essential for oligodendrocytes upon glucose deprivation to break down fatty acid, possibly by utilizing myelin lipids, in order to maintain axon support in an attempt to prevent neurodegeneration [350,351,352]. Elevated very long-chain fatty acids (VLCFA) induce oxidative stress in oligodendrocytes and have been associated with neurotoxicity [353,354]. Hereby, peroxisomes contribute to axon maintenance through beta oxidation of VLCFA and ROS detoxification [350,355,356]. Myelin phagocytosis can trigger anti-inflammatory reprogramming, but excessive lipid uptake, especially of myelin debris enriched in VLCFA, can disrupt lipid metabolism and promote a proinflammatory phenotype, since macrophages and phagocytes in the rim of chronic active white matter lesions do not upregulate peroxisomal genes involved in beta oxidation [353]. Peroxisome deficiency in oligodendrocytes causes axonal degeneration, demyelination, and neuroinflammation [355]. Furthermore, peroxisomal transcripts are reduced in the gray matter neurons and white matter macrophages and oligodendrocytes in MS brains, further contributing to these processes [357,358].

Increased fatty acid metabolism can contribute to ROS production and oxidative stress [359]. Saturated fatty acids promote microglial activation and inflammation, while proinflammatory stimuli in microglia promote saturated fatty acid synthesis [360,361,362,363]. Ceramide-enriched exosomes released from stressed, cytokine-induced oligodendrocytes as well as increased ceramide biosynthesis in reactive astrocytes mediate oligodendrocyte cell death [364,365]. On the contrary, ceramides may also trigger further immune responses by enhancing the effect of Th1 cytokines [364]. Remarkably, increased ceramide levels (C24 and/or C16) in the CSF of (P)MS patients can cause mitochondrial dysfunction and bioenergetic failure in neurons in vitro [366,367]. Increased oxysterol levels have been linked to the disruption of the BBB [368,369]. OPCs can both contribute to BBB disruption and associated CNS inflammation in injured white matter, as well as support BBB integrity and control neuroinflammation, as genetic ablation of NG2+ OPCs led to microglial overactivation and neuronal death [370,371,372,373,374,375]. On the contrary, NG2 knockout mice display milder EAE with reduced immune responses [376,377,378]. Remarkably, specific subsets of OPCs/oligodendrocytes display immunomodulatory and/or immunocompetent properties. They express MS susceptibility genes (e.g., interferon responsive genes), MHC-I genes thereby directly attracting cytotoxic CD8+ T cells, or Ifng-induced MHC-II genes thereby promoting CD4+ T cells. Some are capable of phagocytosis, even of myelin, and express various cytokines and chemokines [267,379,380]. Furthermore, oligodendrocyte death is sufficient to trigger an adaptive autoimmune response against myelin resulting in extensive myelin and axonal loss [381]. Myelin sheaths may be prone to generating antigenic components [382,383,384]. Citrullinated MBP is highly immunogenic, in particular through the MS-associated HLA-DR15 haplotype and can induce Th17 differentiation in CD4+ T cells of healthy controls [385]. Citrullinated Mbp also causes EAE given that encephalitogenic T cells preferentially react with it [386].

Finally, iron metabolism in OPCs and oligodendrocytes is necessary for oxygen utilization, for enzymes involved in ATP production, cholesterol and lipid synthesis thereby promoting OPC differentiation and myelination [387,388]. Iron accumulation in brains is age-dependent, and further enhanced in MS patients, in particular with advanced phenotypes, and slightly correlates with cognitive impairment [389,390,391]. Iron accumulates, in decreasing order, in oligodendrocytes, macrophages/microglia, and astrocytes [392]. However, intracellular iron is cytotoxic when it exceeds the storage capacity of ferritin and induces cell death, and ferroptosis (an iron-dependent lipid peroxidation under glutathione insufficiency), thereby impeding remyelination [270,271,393,394]. Moreover, redox active ferrous iron (II/Fe^2+^) accumulates in the intracellular and extracellular space (especially in the rim of chronic lesions and in the vicinity of active lesion sites), released from harmed oligodendrocytes and myelin debris, and is oxidized to ferric iron (III/Fe^3+^). This produces hydroxyl radicals, alongside the increase in lipid peroxidation and the reduction in antioxidant pathways, leading to ferroptosis [395]. The uptake of oxidized iron by microglia causes their dystrophy and a second wave of iron (II) release [152,270,271,396,397,398]. Therefore, iron deposition and release exacerbate the oxidative stress, creating a noxious environment for other cells and subsequent axonal/neuronal injury. This further contributes to the maintenance of a low-grade chronic inflammation by promoting a proinflammatory microglial polarization, as suggested by the formation of a paramagnetic rim in smoldering lesions [152,399,400]. Moreover, the iron load is higher in the basal ganglia and thalamus, two regions where gray matter atrophy occurs early in MS [389,401]. Both correlate with cognitive impairment, albeit iron accumulation to a lesser extent [391].

## 4. Neurodegeneration in MS

Neurodegeneration starts at onset of disease as evidenced by atrophy and widespread abnormalities on brain MRI, both in white and gray matter, and is not only restricted to lesions. Extra-lesional gray matter changes more consistently correlate with disability [146,163,402,403]. Although it may initially result from Wallerian degeneration in periplaque white matter, distal of earlier acquired white matter lesions, it further develops independently of the extent of active disease, i.e., the total white matter lesion load, but is rather linked to mechanisms of intrinsic neurodegeneration or subsequently driven by diffuse smoldering microglial activation and meningeal lymphocytic inflammation [10,33,144,152,159,164]. Axonal and neuronal injury may even occur without demyelination [97,404,405].

Axonal injury correlates better with the patient’s permanent neurological deficits [406,407,408,409]. Neurodegeneration is the greatest determinant of the risk and latency to disease progression, which occurs once the compensatory capacity of neuronal injury is exceeded and hence depends on the patient’s age rather than the number of relapses, explaining why DMTs have a more modest impact on disease progression [6,21,23,26,410] (Figure 3 and Figure 4, Supplementary Table S1).

### 4.1. Self-Sustained

Early gray matter neurodegeneration occurs, mainly driven by local failure of trophic and anti-inflammatory cellular interactions, despite the upregulation of myelination pathways, as evidenced by spatial transcriptomics and high-sensitivity proteomics performed on cortical brain tissues of SPMS patients vs. controls. Remarkably trophic interactions were already reduced in intact gray matter of MS patients as compared to controls but further reduced in degenerating MS gray matter [411]. Basal autophagy and mitophagy ensure cellular homeostasis; both their loss and excess cause neuronal cell death [412,413,414].

Axonal transection in white matter lesions, occurring early in the disease course, causes Wallerian or anterograde (downstream) and retrograde (upstream) axonal degeneration, thus at a distance from the site of initial injury [164,415]. Moreover, axonal/neuronal injury can also anterogradely or retrogradely induce the transsynaptic degeneration of a synaptically connected neuron, which may occur both in demyelinated and in normal appearing tissue in MS [416,417,418]. At a distance from an acute attack of optic neuritis, the atrophy of the thalamus and primary visual cortex is accelerated (suggesting anterograde transsynaptic degeneration), while the ganglion cell-inner plexiform (GCIP) layer and the inner nuclear layer of the retina are thinner (suggesting retrograde transsynaptic degeneration) [417]. Moreover, the magnitude of tissue injury, the reduction in the GCIP layer, and thalamic atrophy were positively correlated. Furthermore, thalamic and subcortical gray matter atrophy and visual outcome were also correlated [417]. Transsynaptic degeneration may thus signify a more aggressive primary demyelinating event, poorer tissue repair, or remyelination failure [416,417,419]. Remarkably, even patients without a history of optic neuritis show a thinning of the retinal layers, suggesting that lesions on the optic radiation can impact the retina [419].

Paranodes can be elongated and disorganized on myelinated axons at the border of chronic lesions, and thereby contribute to axonal degeneration and subsequently threaten further myelin loss [420]. Focal axonal damage can occur with intact myelin sheaths, initiated by intra-axonal mitochondrial pathology, resulting in an axonal energy deficit and major ion imbalance, mainly of sodium and calcium [219]. Calcium overload, exacerbated by sodium pump deficiency and excessive glutamate release, critically contributes to axonopathy [421]. Sodium accumulates in the axoplasm, and is replaced by calcium, due to the decreased activity of the sodium/potassium ATPase, and the reversing of the sodium/calcium exchanger [214,422,423,424]. Increased sodium channel Nav1.2 activity exacerbates neuroaxonal degeneration independently of immune cell infiltrates [425]. Inhibition of axonal voltage-gated sodium channels prevents mitochondrial morphological changes induced by oxidative stress and preserves the mitochondrial membrane potential [422]. Alteration in glutamate levels also results in calcium influx through extrasynaptic or overactivated presynaptic NMDA receptors, and calcium-mediated excitotoxicity [232,426,427]. Respiratory chain deficits further compromise mitochondrial calcium storage via mitochondrial calcium uniporter (MCU), critical for axonal survival, resulting instead in fragmented mitochondria and autophagosomes. Hence, in neuronal MCU-deficient mice with EAE mitochondrial dysfunction, myelin loss, axonal injury, and inflammation were elevated while remyelination was suppressed [428]. However, this relies on a very tight balance since mitochondrial calcium overload secondary to MCU overexpression can also induce neuronal cell death [429]. Likewise, MCU inhibition under oxidative stress conditions prevents the decrease in mitochondrial motility and preserves the membrane potential [422]. Furthermore, the monocationic TRPM4 channel (transient receptor potential cation channel subfamily M member) is activated by high intracellular calcium levels, while it is impermeable to it, and blocked by high cytosolic ATP levels, thereby colocalizing with axonal injury, while its inactivation reduced neuronal/axonal degeneration [430,431]. Proton-gated acid-sensing ion channels (ASIC) are activated by inflammation-linked tissue acidosis, allowing excessive calcium and sodium influx which subsequently leads to neurodegeneration. The increased expression of ASICs in oligodendrocytes and axons within lesions of EAE mice has been linked to axonal and myelin damage. In Asic1a-deficient mice or mice treated by amiloride, neurodegeneration is reduced independently of lymphocyte/myeloid infiltration [432,433].

As a result, neuroaxonal cytosolic calcium levels increase and activate calcium-activated neutral proteases such as calpains, inducing the proteolytic degradation of cytoskeletal proteins, structural axonal damage, and the disruption of axonal transport, microscopically seen as axonal swelling [214,423,428,434,435,436]. This leads to a vicious circle that further compromises mitochondrial function and energy production, although axonal redistribution of sodium channels, voltage-gated calcium channels, acid-sensing ion channel (ASIC1a), and/or TRPM4 is initially an attempt to preserve axonal conductance and integrity [33,333,424,425,431,432,433]. Moreover, calcium overload activates the mitochondrial permeability transition pores, resulting in the release of mitochondrial solutes (up to 1500 kD, of which cytochrome c), matrix swelling, membrane disruption, and thus ultimately mitochondrial collapse and cell death [437,438,439].

### 4.2. Impacting Neuroinflammation and Demyelination

Chronically demyelinated axons may not be receptive to remyelination given that their radial ensheathment was found to be rare even though premyelinating oligodendrocytes extended processes to demyelinated axons in chronic lesions [440]. Moreover, a primary axonal insult could potentiate acute oligodendrocyte loss by lack of axonally-derived growth factor and drive secondary demyelination [331,441]. Synaptic dysfunction impacts OPC differentiation, as OPCs have electrical properties (via sodium channels, glutamate/GABA receptors) and sense synaptic inputs from neurons within neuron-to-OPC synapses [442,443]. Furthermore, bidirectional neuronal–microglial communication is involved in synaptic transmission. Neuronal ATP release secondary to NMDA receptor activation triggers microglial process outgrowth, while microglia can affect synaptic activity and plasticity [444,445].

Finally, ion imbalance in axons further contributes to microglial activation and neuroinflammation subsequently reinforcing neurodegeneration. Sodium channel Nav1.6 is upregulated in microglia and macrophages in EAE and MS and contributes to their activation and their phagocytic properties [446]. The increased activity of neuronal voltage-gated Nav1.2 channel (by a gain of function mutation) in transgenic mice with EAE exacerbated inflammation-induced neurodegeneration, irrespective of immune cell alterations [425].

## 5. Triangulation in Other Neurodegenerative Disorders

Both Alzheimer’s (AD) and Parkinson’s disease (PD) are neurodegenerative disorders deeply linked to age wherein protein aggregates, extracellular amyloid beta plaques, and intracellular neurofibrillary tau tangles in AD, alpha-synuclein in intraneuronal Lewy bodies in PD, accumulate in distinctive areas, the hippocampus and neocortex in AD, the substantia nigra (pars compacta) and striatum in PD. These aggregates disrupt metabolic processes, leading to neurodegeneration via a common denominator that is neuroinflammation [30]. In fact, there is also the toxic accumulation of a protein in MS, namely of synaptic protein Bassoon, in the neuronal cell bodies, induced by neuroinflammation, while its genetic ablation prevents inflammation-induced neuro-axonal injury in mice [447].

**Figure 5:**
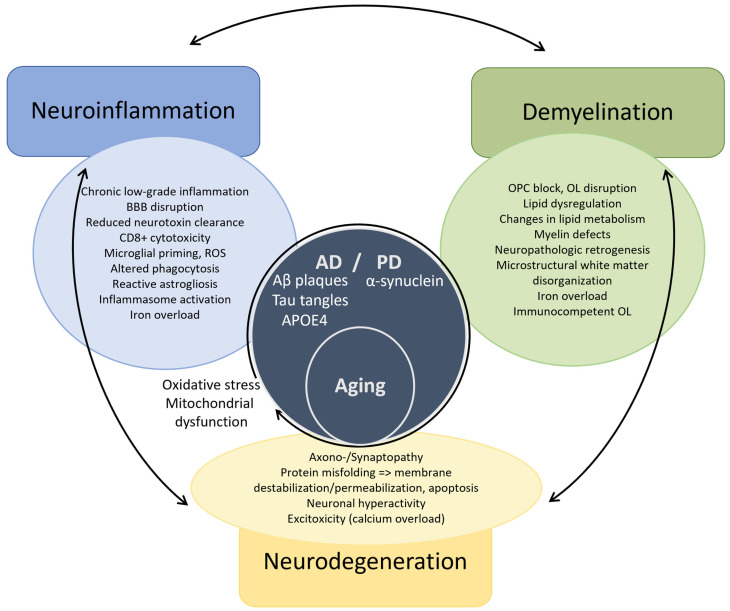
Triangulation of neuroinflammation, demyelination, and neurodegeneration in AD/PD pathogenesis with key mechanisms that self-sustain these processes but also influence each other mutually. The bubbles list pathophysiological mechanisms by which each process (with corresponding color) impacts at least one of the two other processes, while the bubble in the middle highlights peculiar pathophysiological mechanisms involved in all processes. AD = Alzheimer’s disease, PD = Parkinson’s disease, Aβ = amyloid beta, BBB = blood–brain barrier, OPC = oligodendrocyte progenitor cell, OL = oligodendrocyte, ROS = reactive oxygen species.

First, aging is marked by a chronic inflammatory state, with increased proinflammatory mediators and oxidative stress, which is accompanied by BBB disruption at the cellular and molecular level manifesting by its increased permeability. However, the removal of certain neurotoxic substances, such as amyloid beta, is lessened given the reduced expression of efflux transporters [448,449]. The aged brain microenvironment induces microglial priming towards an activated phenotype by itself [450,451,452]. Moreover, cellular senescence in microglia, due to continuous mitotic urge, alters their proliferative response and shifts them towards a proinflammatory phenotype [452,453]. There is transcriptional evidence of a decrease in OPCs and in particular differentiating OPCs in the aging brain, in part due to inflammatory factors released by activated microglia [454,455]. Advanced age is associated with gray matter atrophy due to neuronal loss and with neuroglial functional alterations due to cellular senescence [456,457]. Moreover, continuous white matter deterioration, reflecting demyelination, and axonal damage results in its microstructural disorganization [458,459]. Gray matter volume thereby inversely correlates with increased levels of circulating proinflammatory cytokines, namely TNFa and IL1b, in non-demented elderly subjects [460] (Figure 5, Supplementary Table S1).

### 5.1. Neuroinflammation

Long-term chronic inflammation breaks down beneficial defense mechanisms. Additionally, the inflammatory microenvironment and senescence induce neurotoxic microglia, producing IL1b, TNFa, induced nitric oxide synthase (iNOS), ROS. This results in BBB breakdown and amplifies a peripheral and central inflammatory response and oxidative stress, disrupting brain homeostasis and causing neuronal damage [461,462,463,464,465,466]. Changes in the microenvironment, excessive accumulation of amyloid beta plaques in AD, and the presence of alpha-synuclein in PD, both potent activators of the immune system, enhance cellular senescence processes (DNA damage, accelerated telomere shortening, slowed cell cycle) and lead to microglial activation and astrogliosis. This induces the activation of the NLRP3 inflammasome, which itself activates microglia/astrocytes, and the release of proinflammatory cytokines and oxidative molecules [464,467,468,469,470,471,472,473,474,475]. Microglial NLRP3 activation induced by amyloid beta promotes tau hyperphosphorylation and aggregation, which can subsequently activate microglial NLRP3 as well [476,477]. Microglial phagocytosis of protein aggregates is impaired [478,479,480]. Oxidative and endoplasmic reticulum stress further accelerates protein misfolding, causing cellular damage and mitochondrial dysfunction [481]. Mitochondrial dysfunction with enhanced ROS production in microglia in turn triggers the activation of the inflammasome [482,483,484]. This vicious circle sustains neuroinflammation, hallmarked by a dramatic response in primed microglia, which, combined with age-related systemic inflammation, amplifies neurodegeneration and promotes disease progression [464,485,486].

Three amyloid beta-reactive microglia subpopulations have been described in AD. A first subpopulation, with three distinctive states hallmarked by the expression of the transmembrane glycoprotein nonmetastatic melanoma protein B (GPNMB), displays neuroinflammatory alterations early in the process of amyloid beta plaque deposition, characterized by the upregulation of genes involved in autophagy, antigen processing, and presentation or cytokine response [487]. A second microglial subpopulation, called white matter-associated microglia (WAM), responds to myelin debris and contains MBP+ intracellular particles [488]. Finally, damage-associated microglia (DAM) are characterized by the downregulation of homeostatic genes (e.g., purinergic receptor P2RY12), which correlates with neuronal cell loss, and the upregulation of DAM genes, such as apolipoprotein E (APOE) and triggering receptor expressed on myeloid cells 2 (TREM2) [489,490,491]. TREM2 is crucial to the metabolic reprogramming of microglia and the maturation of DAM. However, inappropriate DAM activation is deleterious [492]. TREM2 induces an APOE pathway that mediates a switch from homeostatic to neurodegenerative microglia [493,494]. On the contrary, TREM2 risk variants for late-onset AD exhibit a reduced function and TREM2 inactivating mutations cause lethal early-onset dementia (i.e., Nasu–Hakola disease) [492,495]. TREM2 deficiency impairs microgliosis (in particular clustering around amyloid beta plaques), enhances their autophagy and apoptosis, and causes axonal dystrophy [496,497,498]. Moreover, while acute exposure to amyloid beta enhances microglial phagocytosis, it regresses with chronic exposure [499]. Increased systemic inflammation impairs microglial amyloid beta clearance in mice via mechanisms mediated by the NLRP3 inflammasome [500]. APOE4, a risk factor for sporadic AD, supports a stronger proinflammatory reaction and causes the breakdown of the BBB by activating a proinflammatory cascade (mediated by cyclophilin A) resulting in the neuronal uptake of blood-circulating neurotoxins, all of which subsequently contribute to neurodegeneration [501,502]. Finally, AD astrocyte clusters are involved in synapse organization, cytoskeletal and extracellular matrix organization, in acute inflammatory response, oxidative and glutamate signaling, in apoptotic signaling due to DNA damage, and may also lose their neuroprotective roles at a transcriptional level [503]. Reactive astrogliosis has been evidenced by increased GFAP plasma levels in the early, preclinical stage of AD, and correlates with neuronal injury (neuritic plaques, amyloid beta plaques, and neurofibrillary tangles) and the clinical onset of cognitive impairment [504,505].

Likewise in PD, alpha-synuclein aggregation may result from a proinflammatory state, a compromised protein-folding machinery and reduced proteolytic abilities linked to aging [506,507]. Single-nucleus RNA sequencing of human postmortem midbrain tissue in PD revealed (i) reactive astrogliosis and (ii) activated microglia, both characterized by the expression of heat shock proteins, (iii) dysfunctional dopaminergic neurons, and (iv) a decrease in myelinating oligodendrocytes that are moreover transcriptionally stressed. This is accompanied by the stress-induced upregulation of the unfolded protein response pathways, in particular detrimental to neuron survival [508,509,510]. Increased levels of IL1b, IL6, TNFa, IFNg, and TGFb1 in the striatum of neurotoxin-treated aged mice as well as a higher density of microglia and forward cytotoxic CD8+ T cells in the substantia nigra have been linked to the decreased density of dopaminergic neurons [511,512,513]. The proinflammatory response in macrophages and microglia is mediated via the JAK/STAT (Janus kinase/Signal transducer and activator of transcription) pathway, resulting in the expression of iNOS, IL6, TNFa, MHC-II. Notably, pharmacological inhibition of JAK/STAT in a rat model of PD overexpressing alpha-synuclein suppressed microglial activation, macrophage, and T cell infiltration and the expression of proinflammatory mediators while it prevented the degeneration of dopaminergic neurons [514].

However, similar to MS, peripheral immune dysregulation might play a role as a pathogenic trigger in AD and PD, inducing neuroinflammation and consequential neuronal damage resulting in motor and cognitive impairment [30]. Clonally expanded T cells have been identified both in AD and PD brains [515,516]. AD may start with a sequence of immunological events. Several serum cytokines (IL1b, IL2, IL4, IL10) are elevated [517]. In particular, cytokines such as IL1b, IL6, TNFa, IFNg, and TGFb can induce gamma-secretase enzymatic activity through the Jun N-terminal kinase (JNK) pathway, resulting in the cleavage of amyloid beta precursor protein and amyloid beta formation [518]. Amyloid beta further activates glial cells [467]. In PD, peripheral immune cells (monocytes, T cells) might initially react to alpha-synuclein primarily misfolded in the olfactory bulb or enteric nervous system and propagating then transsynaptically from nerve cell to nerve cell via the gut–brain axis and the vagal nerve [519,520,521,522]. Moreover, CD8+ T cell infiltration precedes alpha-synuclein aggregation and neuronal cell death [511]. Peripheral inflammation further activates microglia prior to neurotoxic astrocytes and dopaminergic neuron loss in a PD mice model [523]. BBB disruption and peripheral inflammation potentiate neuroinflammation and the degeneration of nigral dopaminergic neurons in animal models [524,525,526].

Finally, iron levels are elevated in brain regions affected by neurodegeneration, namely in the frontal, parietal, and temporal lobe, the amygdala, the cingulate cortex, globus pallidum, putamen, and caudate nucleus in AD and in the substantia nigra and the caudate nucleus in PD [527,528,529]. In PD, iron was increased in microglia and dopaminergic neurons and was closely linked to microgliosis [529]. In AD, the iron load is less dramatic than in PD despite neuroinflammation. The correlation with neuroinflammation is still unclear, but iron may contribute to amyloid beta formation [530,531] (Figure 5, Supplementary Table S1).

### 5.2. Demyelination

Although demyelination is not at the forefront of AD and PD, white matter degeneration is known to occur in these diseases, even in preclinical stages, and can contribute to disease progression [32,532,533]. Moreover, misfolded proteins contribute to oligodendrocyte disruption through lipid dysregulation and organellar stress [31].

Remarkably, the areas more vulnerable to AD pathology have the most protracted and extended course of myelination. The latest myelinated brain regions degenerate first (called neuropathologic retrogenesis), which was also evidenced in PD [534,535,536]. White matter hyperintensities (on brain MRI), reflecting mainly small vessel disease and inflammation, predict incident AD and the rate of cognitive decline. They correlate with CSF amyloid beta levels and are associated with genetic risk factors for AD [537,538,539,540,541,542]. Intracortical myelin density is decreased and is not limited to the vicinity of plaques [543]. Alteration in myelin content even precedes amyloid beta plaque and tau tangle pathology [544]. Hereby, the decrease in myelin structures on quantitative MRI measures is negatively correlated with the CSF concentration of tau and amyloid beta in patients at risk of AD, yet without cognitive symptoms [534]. Myelin defects in AD mouse models trigger the production of amyloid beta and the cleavage of cortical amyloid precursor protein. Moreover, although successfully induced, DAMs preferentially clear damaged myelin rather than amyloid plaques [543]. Oligodendrocyte damage in AD is related to (i) amyloid beta-induced cytotoxicity, with in particular elevated levels of soluble amyloid beta in the white matter, (ii) intracellular glial fibrillary tangles formation, while white matter lesions are associated with cortical hyperphosphorylated tau and may be due to Wallerian degeneration, (iii) iron release from myelin breakdown which promotes amyloid beta oligomerization, (iv) hypoxic insult fostered by cerebrovascular pathology, (v) excitotoxicity and intracellular calcium accumulation, and (vi) excessive age-related DNA damage [532,545,546,547,548,549,550,551,552,553,554,555,556]. Single-cell RNA sequencing on the postmortem prefrontal cortex of AD patients identified oligodendrocyte clusters with upregulation of axonogenesis, synapse organization, and cholesterol metabolism vs. an oligodendrocyte cluster with downregulation of synapse transmission, ion transmembrane transport, and metabolism [503]. Oligodendrocyte transcriptional signatures reflected impaired axonal myelination alongside metabolic adaptation to neuronal degeneration [494]. Moreover, APOE4 isoform altered intracellular lipid homeostasis resulting in increased unsaturated fatty acids in induced pluripotent stem cell-derived glia [557]. Oxidative stress, induced by amyloid beta, reduces the expression of genes promoting OPC differentiation [558]. Amyloid beta aggregates trigger senescence in OPCs [559]. Oligodendrocytes are less functional in the human precuneus in early AD [489]. Glycolytic defects in the oligodendrocyte can induce the assembly of the NLRP3 inflammasome and pyroptotic oligodendrocyte cell death [560].

In PD, dopaminergic neurons of the substantia nigra are unmyelinated or lightly myelinated and thus lack the basal support of the oligodendrocyte in case of increased energy requirements [561]. Moreover, alpha-synuclein preferentially aggregates in unmyelinated axons, enhancing their vulnerability to external stressors and degeneration [533]. However, regional white matter hyperintensities (on brain MRI) are associated with motor deficits, possibly independently of dopaminergic neuron loss [562]. Hereby, accumulation of alpha-synuclein in motor tracts was associated with an increase in the density of OPCs and an enlargement of mature oligodendrocytes but a decrease in myelin proteins alongside a progressive disorganization of white matter axon (scattered alignment) [563]. Oligodendrocytes generated from patient-induced pluripotent stem cells show a delayed maturation, but an increased expression of MHC genes [564]. Cell-type-specific signatures in the PD cingulate cortex/substantia nigra revealed that OPCs/oligodendrocytes were predominantly affected within metabolic processes, gene regulation, and cell differentiation [565,566]. Diffusion tensor imaging further supports white matter disruption in the cingulum of PD patients [567]. Furthermore, cognitive impairment in PD is associated with abnormalities of (pre-)frontal and interhemispheric white matter tracts, rather than with gray matter atrophy [568,569]. Alpha-synuclein inhibits oligodendrocyte maturation and myelination by increasing the content of myelin phospholipids [570].

Moreover, both in AD and PD, oligodendrocytes exhibit a reactive immunocompetent phenotype characterized by the upregulation of complement component C4b in response to amyloid beta and alpha-synuclein exposure, and accompanied by the expression of serine peptidase inhibitor, clade A, member 3N (*Serpina3n*), and proteasome 20S subunit beta 9 (*PSMB9*), respectively [494,564]. Finally, astrocytosis in the white matter is associated with loss of myelin in AD, PD, and normal aging [571].

Finally, sulfatide depletion in relevant brain regions in AD and PD mice models indicates myelin disruption. In PD brains, only long-chain hydroxylated sulfatides were depleted suggesting the contribution of oxidative stress [572,573]. Sulfatide deficiency is sufficient to induce AD-like neuroinflammation by microglial and astrocytic activation (marked by increased expression of AD risk genes) and contributed to cognitive impairment [574]. Furthermore, lysophosphatidylethanolamines and lysophosphatidylcholines, implicated in neuroinflammation via activation of phospholipase A2, accumulate in amyloid plaques [572]. APOE4 isoform alters the metabolism of several lipids and increases cholesterol biosynthesis but decreases its transport in oligodendrocytes, resulting in increased formation of cholesteryl esters with aberrant intracellular storage in lipid droplets and decreased localization to the plasma membrane, causing endoplasmic reticulum stress, and hypomyelination [575]. Both sulfatide depletion and cholesterol alteration are mediated by APOE4 and accelerated by amyloid beta accumulation in AD [572,574,575] (Figure 5).

### 5.3. Neurodegeneration

Protein misfolding triggers neuronal dysfunction and death, mainly by membrane destabilization or permeabilization [576,577,578,579]. Hereby, endoplasmic reticulum stress upregulates the unfolded protein response pathways, in an attempt to degrade misfolded proteins, but prolonged cellular stress eventually leads to apoptosis, in particular, in neurons [508,509,510]. In AD, the abnormal accumulation of amyloid beta as oligomers forms pore-like structures with channel activity in the synapses causing synaptic damage. Alteration in glutamate receptors and signaling pathways, circuitry hyperexcitability, mitochondrial and lysosomal dysfunction further contribute to synaptic and axonal pathology and defective neurogenesis in AD [580]. Synapses are also remarkably vulnerable to synucleopathy, given that the progression of brain atrophy in PD has been linked to the structural and functional brain connectivity in the caudate nucleus, nucleus accumbens, hippocampus, and posterior cortical regions, while it was inversely related to the presence of oligodendrocytes [581].

Dopaminergic neurons may increase their pacemaking and bursting activity in order to compensate for the lost neurons in maintaining dopamine levels but are also more vulnerable due to increased intracellular calcium levels [582,583,584,585]. However, this increases their energetic needs and oxidative stress while mitochondrial function may be hampered (via respiratory chain dysfunction), and can induce excitotoxicity and subsequent intracellular calcium excess [583,586,587,588,589,590]. Reactive astrocytes fail to overexpress glutamate transporters for its turnover [591]. Neuronal hyperactivity and oxidative stress also enhance alpha-synuclein aggregation, release, and spreading [592,593,594]. Increased cytosolic dopamine levels are toxic, as mitochondrial dysfunction promotes dopamine oxidation, and these metabolites then promote lysosomal dysfunction and alpha-synuclein accumulation, further contributing to oxidative stress and mitochondrial dysfunction which are also enhanced by aging processes [595,596]. This damages dopaminergic neurons, that also stimulate microglial activation which subsequently contributes to dopaminergic neuron necrosis [464,467,597,598]. On the other hand, nuclear receptor-related 1 protein (*NURR1*) expression, a transcription factor important in dopaminergic neuron homeostasis and regulation of neuroinflammation, was significantly downregulated in peripheral blood mononuclear cells of PD patients alongside the upregulation of several cytokines (*TNFa*, *IL1b*, *IL6*, and *IL10*) [599].

Finally, neuronal NLRP1 inflammasome activation induces caspase 1 (CASP1)/IL1b-mediated neuroinflammation, CASP6-mediated axonal degeneration, and neuronal pyroptosis in AD [600,601]. CASP1 also contributes to alpha-synuclein cleavage and its subsequent intraneuronal aggregation in neuroblastoma cells in vitro [602] (Figure 5).

## 6. Triangulation in Metabolic Syndrome

Metabolic syndrome, a compilation of central obesity, hypertension, dyslipidemia, and (pre-)diabetes, is accompanied by a chronic low-grade inflammation nourished by the adipose tissue [36,603]. The hypertrophy of the adipose tissue creates a hypoxic environment causing endoplasmic reticulum stress, lipolysis (above liposynthesis), insulin resistance, and cell death in adipocytes, driven by the overactivation of hypoxic inducible factor 1 alpha (HIF1a). This results in the release of damage-associated molecular proteins and in an increase in circulating free fatty acids, both inducing a proinflammatory immune response via Toll-like receptor 4 (TLR4) and downstream Nuclear factor kappa B (NFkB) pathway mediated by macrophages, thereby enhancing the former [36,604,605,606]. Moreover, stressed hypertrophic adipocytes release cytokines (TNFa, IL6) and adipokines (increased leptin, decreased adiponectin) thereby closing the loop of a reciprocal influence [607]. Increased circulating free fatty acid, decreased circulating adiponectin levels, and leptin resistance decrease lipid oxidation in non-adipose tissues, further enhancing lipid accumulation, lipotoxicity and insulin resistance [604,608,609]. Furthermore, obesity contributes to oxidative stress via several mechanisms such as hyperglycemia, hyperleptinemia, low antioxidant defense, chronic inflammation, and post-prandial ROS production [610].

Of note, leptin, a hormone of satiety released by the adipose tissue to regulate anorexigenic-mediated energy balance via its receptors in the hypothalamus, also has neurotrophic properties via receptors expressed in the hippocampus and neocortex. Its opponent, adiponectin, activates anorexigenic neurons in the hypothalamus at low glucose levels and inhibits them at high levels. It has further insulin-sensitizing, anti-inflammatory, anti-apoptotic, and neuroprotective properties [611,612,613].

**Figure 6:**
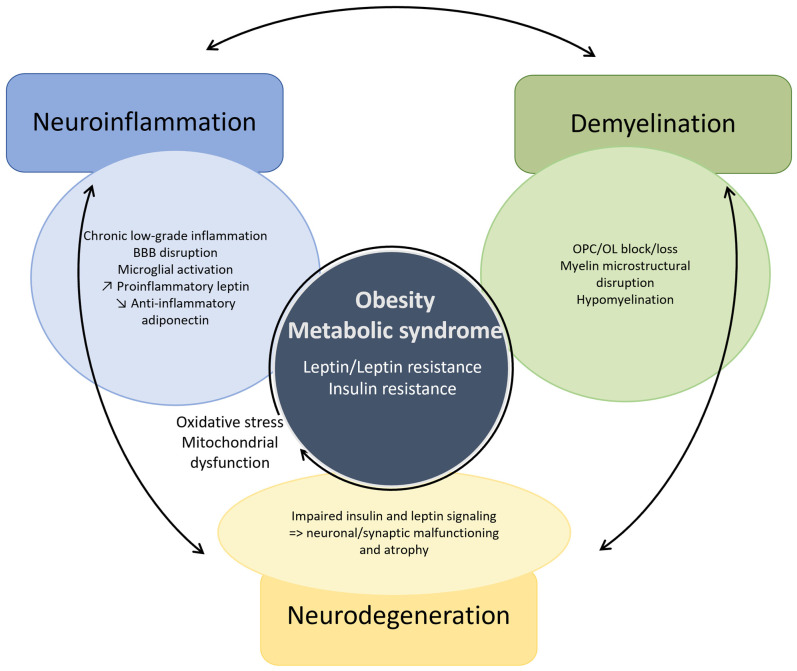
Triangulation of neuroinflammation, demyelination, and neurodegeneration in metabolic syndrome with key mechanisms that self-sustain these processes but also influence each other mutually. The bubbles list pathophysiological mechanisms by which each process (with corresponding color) impacts at least one of the two other processes, while the bubble in the middle highlights peculiar pathophysiological mechanisms involved in all processes. BBB = blood–brain barrier, OPC = oligodendrocyte progenitor cell, OL = oligodendrocyte, ↗ = increased, ↘ = decreased.

Hence, obesity has been linked to neurodegeneration and neurodegenerative disorders as MS, AD, and PD [614,615,616,617,618,619,620]. The mechanisms are multiple but mainly rely on BBB compromise, neuroinflammation, oxidative stress, mitochondrial dysfunction, insulin and leptin resistance causing impaired synaptic plasticity and neuronal death [36] (Figure 6, Supplementary Table S1).

### 6.1. Metabolic Syndrome as Trigger of Neurodegeneration

#### 6.1.1. Neuroinflammation

The peripheral chronic low-grade inflammation instigated by the adipocytes can facilitate the passage of peripheral immune cells through the BBB [621]. Moreover, hyperglycemia-induced oxidative stress in pericytes contributes to BBB disruption [622]. This subsequently causes neuroinflammation, more prominently in certain brain areas rather involved in cognition and memory, such as the cerebral cortex, hypothalamus, and hippocampus [623,624,625,626].

Leptin supports proinflammatory immune responses within the CNS, especially in microglia [627,628,629]. Neuroinflammation and microglial activation contribute to hypothalamic leptin resistance [630]. Oppositely, adiponectin has an anti-inflammatory effect by repressing macrophage and microglial activation via TLR4 and AdipoR1/NFkB signaling and thereby enhancing their anti-inflammatory phenotype, but adiponectin levels decrease in obesity [629,631,632,633].

Furthermore, the diversity of the gut microbiota regulates the BBB and microglial homeostasis and supports normal brain development and functioning via chemical and physical connections mediated by immune, enteric, and neural pathways [634,635,636,637,638]. On the contrary, obesity-induced gut dysbiosis causes microglial activation, among others by the release of various bacterial products in the blood (such as lipopolysaccharide) [639,640] (Figure 6, Supplementary Table S1).

#### 6.1.2. Demyelination

Acute nutrient shift influences OPC proliferation and differentiation, while chronic nutrient shift affects both oligodendrogenesis and myelination. Hereby, undernutrition has a negative impact, and overnutrition has a positive impact on these processes, possibly via insulin, leptin, or thyroid hormones. However, chronic overnutrition accompanied with neuroinflammation, BBB disruption, and brain insulin resistance could halt OPCs and oligodendrocytes, leading to hypomyelination [641,642,643,644]. A chronic high-fat diet in mice promotes the loss of OPCs and oligodendrocytes in the brain and spinal cord alongside transcriptomic and metabolomic changes in endoplasmic reticulum stress, mitochondrial dysfunction, and oxidative stress pathways [645]. It also triggers myelin microstructure disruption and prolonged microglial activation in the hypothalamus [646]. The impact of obesity on the white matter is still disputed; however, a lower myelin content has been linked to obesity in cognitively healthy adults, including the anterior and posterior thalamic radiation, the inferior fronto-occipital fasciculus, the inferior and superior longitudinal fasciculus, the uncinate fasciculus, the corpus callosum, the internal capsule, the cingulate gyrus, the hippocampus, and the corticospinal tract [647,648,649,650] (Figure 6, Supplementary Table S1).

#### 6.1.3. Neurodegeneration

Obesity compromises the gray matter in cognitively normal, young to middle-aged and elderly subjects, as marked by reduced cortical thickness and brain atrophy, especially in the left and right inferior frontal gyrus, the left middle temporal gyrus, the left precentral gyrus, the cingulate gyrus, hippocampus, thalamus, and the left cerebellum [651,652,653,654,655]. This is partially explained by the cerebral hypoperfusion linked to obesity [656,657]. Moreover, hyperglycemia may result in the downregulation of GLUT1 (expressed on brain endothelial cells) and GLUT3 (expressed on neurons) in order to reduce cerebral glucose uptake and its cytotoxicity [658,659,660]. Others showed an increased glucose uptake in the brain with hyperinsulinemia [661,662]. Herein, insulin signaling in hypothalamic astrocytes regulates glucose uptake across the BBB [663]. Insulin receptors are highly expressed in the brain, in particular in the cortex, the hypothalamus, and the hippocampus [664,665]. Insulin is crucially involved in synaptic plasticity and activity, but insulin transport through the BBB is compromised with insulin resistance, impairing its central action [666,667,668,669]. Thus, impaired central insulin signaling enhanced by hyperinsulinemia and insulin resistance affects neuronal and synaptic functioning, thereby impairing memory and learning processes [667,669,670,671,672]. Insulin resistance correlated negatively with gray matter volume in the right medial frontal cortex in healthy controls and in the medial temporal cortex in AD patients [673].

Both leptin and adiponectin promote neuronal proliferation in the hippocampus and synaptogenesis, and modulate post-synaptic signaling [674,675,676,677,678]. Leptin receptors are widely expressed in the brain, including the hypothalamus, hippocampus, and neocortex [679,680]. Obesity is accompanied by hyperleptinemia with leptin resistance (due to impaired leptin receptor signaling and leptin insensitivity at the BBB or an impaired and saturated transport through the BBB) and hypoadiponectinemia [666,668,681,682,683]. While leptin levels are increased in the blood, they appear lower in the CSF [666,684]. Impaired leptin signaling in the CNS and reduced adiponectin levels may contribute to neuronal atrophy in the hippocampus resulting in memory and/or learning ability impairment, especially in AD. However, the involvement of adiponectin is still debated [685,686,687,688,689].

A high-fat diet causes oxidative stress, supported by a decrease in the mitochondrial oxidative capacity, in the brain cortex and even more in synaptic regions, negatively impacting neuronal plasticity. It is associated with neuroinflammation [690]. A high-fat diet further impairs hippocampal synaptic plasticity by inactivating insulin receptor substrate 1 (IRS1) and downregulating glucose transporters GLUT3/GLUT4 [691]. Obesity-induced gut dysbiosis is also involved in hippocampal apoptosis via mitochondrial dysfunction and oxidative stress, as well as gut and systemic inflammation and microglial activation [639] (Figure 6, Supplementary Table S1).

### 6.2. Metabolic Syndrome as Risk Factor of Neurodegenerative Disorders

*In MS*, obesity occurring during adolescence is especially incriminated in increasing the risk of MS. It also potentiates the risk linked to other genetic and environmental factors [619,692,693,694]. Obesity is further associated with an increased risk of conversion from CIS to RRMS, a higher relapse rate and disability burden and progression, a reduced pharmacokinetic response to treatment, as well as cognitive decline and brain atrophy [618,695,696,697,698,699,700,701,702,703,704], although others also found no association with physical and cognitive disability worsening or brain atrophy [705,706,707]. The additional impact of obesity on gut dysbiosis, CNS inflammation, BBB breakdown, and oxidative stress exacerbates disease severity in EAE and MS patients [708,709,710,711,712,713]. Oppositely, caloric restriction could reduce cuprizone-induced demyelination and enhance alternative microglial activation [714]. Herein, the peculiar role of leptin has been highlighted. Leptin drives EAE susceptibility, which can be delayed by acute starvation, and leptin-deficient mice are resistant to EAE [715,716]. Oppositely, treatment with adiponectin ameliorated EAE, while adiponectin deficiency worsened EAE, which was characterized by enhanced neuroinflammation, demyelination, and axonal injury [717]. Leptin levels are increased in both the serum and CSF of RRMS patients and the expression of its receptor is upregulated in CD8+ T cells and monocytes during relapse [718,719]. It increases the proliferation of autoreactive T cells and the production of cytokines, but inhibits Treg proliferation [719,720]. Moreover, active CNS lesions contained high levels of leptin on post-mortem analysis [721].

*In AD*, obesity and diabetes are independent risk factors [614]. A higher body mass index and in particular central obesity in mid-life increases the risk of dementia, while remarkably, in late life, it seems to diminish the burden of the disease [722,723,724,725,726]. A high-fat diet in animal models of AD accelerated cognitive decline due to decreased synaptic plasticity. Underlying mechanisms are BBB disruption, systemic and central inflammation (among which microglial activation), adipokine and insulin signaling dysregulation, altered brain energy metabolism, oxidative stress (partially by reduced activation of redox-sensitive transcription factor Nrf2), and neuronal apoptosis [727,728,729,730,731,732,733,734,735]. Transgenic APP/PS1 mice have chronically elevated basal extracellular and stimulus-evoked levels of glutamate in the hippocampus which are further enhanced by a high-fat diet [736]. A high-fat diet and impaired glucose metabolism also increase amyloid beta deposition and/or tau phosphorylation [730,737,738,739,740]. Moreover, oligodendrocytes treated with palmitate (a saturated fatty acid) enhance insulin resistance in recipient neurons [739].

Late middle-aged persons with insulin resistance already have an increased amyloid deposition in the frontal and temporal areas [741]. Hyperinsulinemia has been associated with a higher risk of AD and promotes the amyloid beta pathology given that (i) insulin increases the secretion of amyloid beta, (ii) it also decreases its degradation given the competition of both insulin and amyloid beta for insulin-degrading enzyme, and (iii) amyloid beta competes with insulin for binding and activation of insulin receptors [742,743,744,745]. Amyloid beta oligomers bind to neurons and cause the dendritic insulin receptors to be redistributed within the cell body, downregulated, and less activated [746]. Insulin signaling pathways are impaired in AD, especially via insulin receptor substate 1 [747]. Increased activation of Glycogen synthase kinase 3 beta (GSK3b) in the brains of AD patients, in particular the hippocampus, accompanies impaired insulin signaling downstream the insulin receptor/insulin-like growth factor receptor through the Phosphoinositide-3-kinase (PI3K)/AKT pathway, resulting in increased phosphorylation of tau and increased production of amyloid beta peptides [747,748,749,750,751,752]. Insulin resistance further impairs glucose uptake and metabolism in the brain by reducing neuronal GLUT expression, thereby contributing to neuronal energy deficit and impaired synaptic activity [753,754,755,756]. Insulin mediates the translocation of GLUT4 to the plasma membrane supporting glucose demand for the activity of hippocampal neurons [757]. On the contrary, effects on GLUT1 and GLUT3 might be indirect via insulin resistance-induced hyperglycemia or during excitatory stress and their downregulation might contribute to neurotoxic tau and amyloid beta oligomer formation [758,759].

Higher cholesterol levels decrease leptin levels and induce the amyloidogenic pathway, which occurs predominantly in cholesterol-enriched lipid rafts via beta- and gamma-secretases [760,761,762,763,764]. Leptin treatment may alter the lipid composition of lipid rafts. Hereby, it can reverse the cholesterol-induced amyloid beta formation by reducing beta-secretase levels and activity. It also increases amyloid beta clearance and degradation by increasing the levels of low-density lipoprotein receptor-related protein 1 and insulin-degrading enzyme and by increasing APOE-dependent amyloid beta uptake, and promotes the alpha-secretase-mediated non-amyloidogenic pathway [764,765]. Leptin further supports hippocampal neurogenesis and is positively correlated with the volume of the right hippocampus [678,766]. Thus, leptin plays a protective role in AD. Its levels are decreased in the blood but increased in the CSF of AD patients [767,768]. However, leptin resistance was evidenced in neurons, given the reduced expression of its receptor, further enhanced by the APOE4 isoform. Remaining leptin receptors were moreover localized to neurofibrillary tangles [686,767]. Furthermore, the Akt-pathway coupled to these receptors is desensitized in the hippocampus by a high-fat diet in adolescent mice [769]. Oppositely, adiponectin levels are increased in the blood but decreased in the CSF of AD patients and colocalizes with tau in neurofibrillary tangles [685,768]. Plasma/CSF adiponectin levels correlated with hippocampal atrophy and poorer cognitive outcome, although only in women regarding the plasma levels [685,689]. Adiponectin protected in vitro human neuroblastoma cells against amyloid beta-induced cytotoxicity due to oxidative stress, by suppressing NFkB activation [770]. Aged adiponectin-deficient mice showed spatial memory and learning difficulties and developed AD-linked processes such as amyloid beta deposition, tau hyperphosphorylation, alongside impaired insulin signaling, microgliosis and astrogliosis, and increased GSK3b activation in the hippocampus and frontal cortex [771]. Intraperitoneal injection of adiponectin in high-fat diet mice could restore glucose metabolism, reduce amyloid beta aggregates, while improving cognitive functions [735].

Finally, obesity-induced cerebral hypoperfusion and endothelial dysfunction, associated with reduced synthesis and increased degradation (due to oxidative stress) of nitric oxide, enhance the production of amyloid beta in turn worsening endothelial dysfunction [772].

*In PD*, it is not yet well established whether a higher body mass index is a risk factor for the disease [616,773]. PD patients with diabetes experience a faster motor progression and cognitive decline [774,775]. A high-fat diet in rodent PD models accelerates the deposit of alpha-synuclein and exacerbates neurotoxicity and neurodegeneration, alongside earlier motor decline and death [776,777,778,779]. A high-fat diet reduces the expression of peroxisome proliferator-activated receptors and of tyrosine hydroxylase, a precursor molecule of dopamine synthesis, in parts of the dopaminergic axis, namely in the substantia nigra and/or ventral tegmental area, which is accompanied by enhanced neuroinflammation, astrogliosis/microgliosis, oxidative stress, mitochondrial and/or peroxisomal dysfunction, as well as the loss of dopaminergic neurons in the substantia nigra [780,781]. A high-fat diet and insulin resistance impair dopamine transmission, given a decrease in the expression and function of presynaptic dopamine transporters and in the expression of postsynaptic dopamine D2 receptors. Herein, high-fat-fed insulin-resistant young adult rats exhibited a blunted dopamine release and clearance [782,783]. Moreover, the insulin receptor is downregulated in the substantia nigra of PD patients as compared to controls [784].

In conclusion, metabolic syndrome is a risk factor for the development of MS, AD, and potentially PD.

## 7. Possible Therapeutic Strategies for the Future

**Figure 7:**
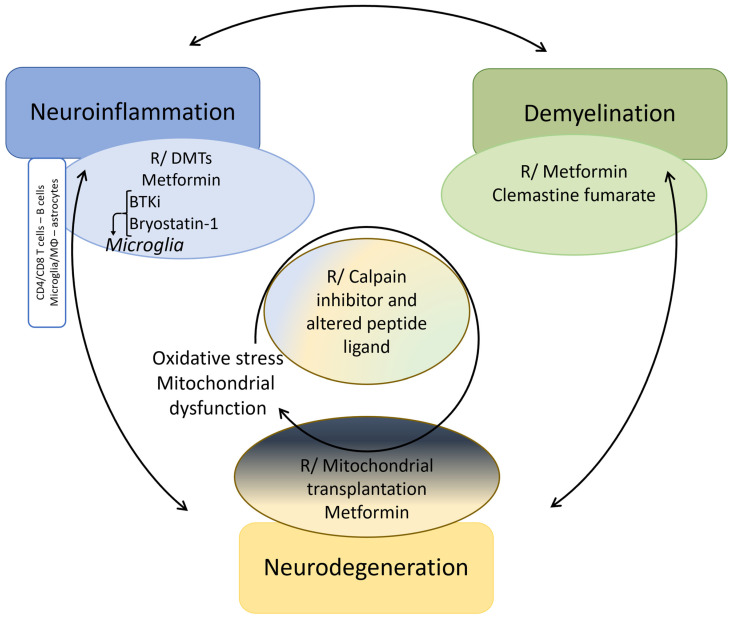
A few novel therapeutic strategies in MS. The well-known DMTs act mainly on the peripheral adaptive immune system by their immunomodulatory properties or by selectively depleting lymphocytes or by impeding their migration. BTK inhibitors and Bryostatin-1 finally add microglia to the panel of therapeutic targets. BTK inhibitors target B cells but could also offer additional benefits by modulating macrophages and microglia. Bryostatin-1 programs microglia/macrophages toward an anti-inflammatory phenotype. Clemastine fumarate promotes OPC differentiation. Metformin has anti-inflammatory properties by reducing microgliosis/astrogliosis and proinflammatory mediators, it reduces oxidative stress, rejuvenizes OPCs, and may enhance neurogenesis. In a yet experimental combination therapy, a calpain inhibitor acts within the 3 facets by reducing myelin loss, axonal damage, and CD4+ T cell expansion, while an altered peptide ligand of MBP alters the effector function of T cells. Finally, mitochondrial transplantation could enhance the number of functional mitochondria in all cell types but would in particular be effective in neurons. BTKi = Bruton’s tyrosine kinase inhibitor, DMT = disease-modifying therapies, MΦ = macrophages, R/ = treatment.

Disease-modifying therapies (DMTs) in MS mainly target the peripheral immune cells. While they can temper the inflammatory component of the disease, they do not directly act on the other components related to disease progression. So far only two DMTs are approved for PMS, i.e., siponimod for active SPMS, and ocrelizumab for PPMS, and seem more effective in younger patients with a shorter progressive phase, when active inflammation is possibly still superimposed [23,26,785,786]. Novel therapeutic strategies are urgently needed. It seems of interest to focus on the pathological mechanisms that are not targeted by current DMTs, such as microgliosis/astrogliosis, oxidative stress, ion imbalance, and remyelination failure [26,787] (Figure 7). Similarly, in AD and PD, therapies aiming to clear amyloid beta plaques and dopaminergic therapies, respectively, are not sufficient to halt disease progression [532,533].

Clinical trials (phase 2 or 3, https://clinicaltrials.gov) are ongoing for Bruton’s tyrosine kinase (BTK) inhibitors in relapsing and/or progressive MS [788]. BTK is a non-receptor tyrosine kinase downstream of the B cell receptor and Toll-like receptor in B cells, participating in their development and maturation [788,789,790,791]. It also mediates microglia and macrophage activation via IgG-specific Fc receptor III and TLR signaling [788]. Its expression level is increased in microglia mainly, to a lesser extent in B cells and astrocytes [792,793,794]. Different from B cell depletion therapy, BTK inhibitors alter B cell function as antigen-presenting cells for the development of encephalitogenic T cells without affecting their frequency and functional integrity [790]. They reduced the severity of secondary progressive autoimmune demyelination in an in vivo mice model and promoted remyelination [794,795]. In particular, Tolebrutinib was recently evidenced to significantly delay confirmed disability progression in patients with non-relapsing SPMS (Hercules study), while it could not reduce the annualized relapse rate in relapsing MS patients (Gemini 1 and 2 studies) [796]. The protein kinase C modulator, bryostatin-1 (phase 1), is able to shift the transcriptional program of microglia and CNS-related macrophages toward a regenerative phenotype supporting phagocytosis and OPC differentiation and preventing the activation of neurotoxic astrocytes [797] (Figure 7). Other yet experimental strategies aim to improve mitochondrial function by restoring the calcium homeostasis or by scavenging peroxynitrite [412,798].

An innovating strategy to rescue energy production and mitochondrial function in injured cells relies on mitochondrial autotransplantation (Figure 7), whereby mitochondria isolated from healthy cells (usually skeletal muscular cells) are administered centrally or systemically in order to be non-specifically incorporated via macropinocytosis in other cells, including the injured cells [799,800,801,802]. Mitochondrial transplantation could ameliorate the clinical outcome of animal models of PD and the cardiac function of four children with cardiac ischemia [803,804,805]. Similarly, enhancing axonal mitochondrial content or activity improved in vitro and in vivo models of MS, for example, by the delivery of functional mitochondria via extracellular vesicles isolated from neural stem cells [806,807,808]. Targeting RNS-mediated excessive autophagy/mitophagy (e.g., by inhibiting peroxynitrite) or inhibiting Kelch-like ECH-associated protein 1 (Keap1) and Keap1–Nrf2 protein–protein interactions in order to enhance the antioxidant Nrf2 pathway are other promising strategies that are currently explored in MS and other neurodegenerative disorders [412,809].

Several remyelinating therapies have already been or are currently being tested, however, with no major breakthrough so far [810]. Clemastine fumarate, a H1-antihistamine inhibiting M1 muscarinic receptors, can promote OPC differentiation and myelination by restoring the non-canonical Contactin 1/Notch1/Deltex 1 signaling pathway and/or by inhibiting the NLRP3 inflammasome pathway and subsequent pyroptosis while enhancing antioxidant mediators (Nrf2 and HO1) (Figure 7). It was shown to suppress microglial and astrocytic activation as well [811,812,813]. Phase 2 and 3 trials in MS are still ongoing [814,815,816]. Moreover, in an AD mouse model, it could decrease amyloid beta deposition, and increase densities of OPCs, oligodendrocytes, and myelin possibly by preventing OPCs from entering in a state of cellular senescence [817]. A clinical trial on opicinumab, an antibody against LINGO1 (leucine-rich repeat neuronal protein 1, a surface protein on OPCs inhibiting their differentiation) in patients with relapsing MS did not reach the primary endpoint (i.e., multicomponent disability improvement over 72 weeks) and its development was thus halted, although outcomes may have been assessed too early following the treatment. Moreover, remyelination therapies will probably be more effective in supporting the remyelinating capacity when administered early, given that opicinumab showed better results in younger patients with shorter disease duration [818,819].

Metformin is largely used in the treatment of type 2 diabetes. It reduces the endogenous glucose production in the liver, as well as the net intestinal glucose uptake by increasing the anaerobic glucose metabolism in enterocytes, resulting in reduced blood glucose levels [820]. Metformin was also found to reduce the risk of cognitive impairment in patients with type 2 diabetes, but an increase in the risk has been evidenced by others [821,822,823]. Metformin mainly acts by activating the AMP-activated kinase and subsequently suppressing NFkB, which acknowledged its anti-inflammatory properties. In MS patients with metabolic syndrome treated with metformin, MBP-reactive cells secreting IFNg and IL17 were reduced, while Tregs were increased in number and regulatory function [824]. In cuprizone-treated mice, it alleviated microgliosis and astrogliosis in the corpus callosum alongside the downregulation of proinflammatory genes without affecting anti-inflammatory genes except of Trem2 [825]. It also reduced demyelination and apoptotic signaling cascades while it enhanced oligodendrogenesis (from the recruitment of OPCs to the differentiation in mature oligodendrocytes) by decreasing the oxidative stress and maintaining ATP levels in oligodendrocytes via induction of antioxidant Nrf2 and inactivation of mechanistic target of rapamycin kinase (mTOR), respectively, subsequently to AMP kinase activation. Thus, metformin has proregenerative effects on OPCs [826] (Figure 7). Hereby, both metformin treatment and alternate-day fasting enhanced remyelination in aged rats. On a cellular level, metformin mimics fasting, reversing certain changes of aging in OPCs, thereby restoring their regenerative capacity and creating a permissive environment for remyelination [827]. Metformin treatment started at the time of EAE induction reduced microglia count, decreased dysmyelination, and improved functional outcomes, while treatment started upon presentation of disease symptoms failed to do so [828]. In a clinical trial on metformin as adjuvant therapy to interferon beta in RRMS patients, it demonstrated a potential effect in reducing malondialdehyde, an oxidative stress marker, but not on any other immunological, MRI, and clinical outcome. However, this was assessed after 6 months of therapy only [829]. Several clinical trials evaluating the benefit of metformin in promoting remyelination and impeding neurodegeneration are ongoing. Regarding AD, patients with mild cognitive impairment, with or without diabetes, and treated with metformin had a better cognitive outcome compared to the respective untreated group alongside reduced thinning of cortical thickness [830]. Metformin could also prevent amyloid plaque load (by reducing beta-secretase expression) and tau phosphorylation (by inducing protein phosphatase 2A or reducing mTOR complex 1) and spreading of tau pathology in respective transgenic mice models [831,832,833]. It reduced neuroinflammation (microgliosis/astrogliosis and proinflammatory mediators) and enhanced neurogenesis in the hippocampus and the cortex in vivo, and hereby improved cognitive functions [831]. It also restored mitochondrial function and insulin sensitivity in neurons in vitro [834,835]. In PD rodent models, it prevented alpha-synuclein phosphorylation, dopaminergic neuronal loss, and improved motor functions [836,837]. Some reported a reduced risk for PD by metformin therapy in combination with sulfonylurea, while others did not, or even showed an increased risk for PD by long-term metformin therapy in patients with type 2 diabetes [823,838,839,840]. Given these controversial results, more studies are necessary to identify patients’ characteristics that may predict a beneficial impact of metformin therapy.

The results of several in vivo studies have prompted the evaluation of novel therapeutic strategies targeting microgliosis/astrogliosis, oxidative stress and/or promoting remyelinating/regenerative processes. Clinical trials have been launched for some, and already arrested for others. However, some elements might be important to take into consideration. The CNS might be less accessible to a potential drug, highlighting the need for an efficient CNS delivery strategy. Research is growing in biotechnology systems, such as nanoparticles or carrier peptides, as it may grant in the future an efficient and targeted drug delivery to the cells of interest with increased solubility, stability, and BBB penetration, with sustained release and the possibility of targeted transfer of combination therapies [806,841,842,843]. Neurodegeneration is a slow process that takes years and begins long before the first clinical symptoms, stressing the need for early intervention. Moreover, the best parameter to assess and follow up neurodegeneration in clinical trials is still unclear (for example, thalamic volume on brain MRI, measurements of macular ganglion cell layer and retinal nerve fiber layer by optical coherence tomography) [844,845]. In addition, clinical trials may miss a clinical effect depending on the stage of the disease when patients are enrolled and the duration of treatment. Since neurodegeneration starts early in the disease and intertwines with neuroinflammation and demyelination, especially in MS, changing strategies to target concomitantly these different pathological mechanisms early in the disease in order to prevent further neuronal damage appear to be an absolute necessity. It might be more relevant to consider a combination therapy with an immune-modulating (both peripheral and central) and a promising remyelinating/neuroprotective agent, which should be started early on and continued for a sufficient duration with relevant multiparametric outcomes measured. For example, a combination treatment with a calpain inhibitor and a novel protease-resistant altered small peptide ligand that mimics MBP improved EAE more strongly than each treatment separately. Altered peptide ligands are analogs of immunogenic peptides in which T-cell receptor contact residues have been altered, perturbing the effector function of T cells. Calpain inhibitor is neuroprotective by reducing myelin loss and axonal damage, and anti-inflammatory by reducing CD4+ T cell expansion while the altered MBP peptide ligand attenuates Th17 cells and increases myeloid suppressor cells and Tregs [846] (Figure 7). 

## 8. Conclusions

Neurodegeneration is a major component of chronic CNS disorders, but also beyond, in chronic disorders with increased systemic inflammation, such as obesity and the metabolic syndrome. Even though neurodegenerative disorders greatly differ in their etiology, pathogenesis, disease course, and CNS topography, they share several pathophysiological mechanisms. Hereby, neurodegeneration alongside neuroinflammation and demyelination drive disease pathogenesis by sustaining themselves and each other through underlying mechanisms such as inflammation and microglial activation, oxidative stress, ion imbalance and energy deficit, mitochondrial dysfunction, excitotoxicity, iron accumulation, virtual and tissue hypoxia, loss of trophic support, myelin alterations, and impaired axonal transport. The connections between these mechanisms are complicated and remain to be fully elucidated. However, it also shows the urge to rethink therapeutic strategies in order to address these processes simultaneously and early on to prevent the subsequent manifold vicious circles underlying these devastating diseases.

### Perspectives

This review elucidates critical issues concerning the interconnected mechanisms of neurodegeneration, neuroinflammation, and demyelination, particularly in the context of diseases such as MS, AD, and PD. Several directions are proposed to address these issues more effectively.

Firstly, additional mechanistic research is required to examine the interplay of mitochondrial dysfunction and oxidative stress in both neurons and glial cells to elucidate new molecular targets, to investigate the role of OPCs in remyelination failure and explore methods to enhance their differentiation and function, and finally to further investigate how systemic factors such as metabolic syndrome exacerbate neuroinflammation and neurodegeneration.

Secondly, there exists a critical need for biomarker development for the early detection of neuroinflammatory and neurodegenerative processes, enabling timely intervention prior to significant damage occurring. Advanced imaging techniques could be implemented to monitor the progression of diffuse white matter and gray matter damage.

Thirdly, cross-disease insights, as revealed in this review, could leverage the shared pathways in MS, AD, and PD to develop treatments targeting common mechanisms such as protein aggregation, iron metabolism dysregulation, and senescence-driven inflammation. Immunomodulatory therapies should be refined to minimize adverse effects while targeting the chronic inflammation central to these diseases. This could include therapies aimed at converting proinflammatory microglia and astrocytes to anti-inflammatory phenotypes. Further research is necessary to investigate how environmental and lifestyle factors such as diet, exercise, and stress management influence neurodegenerative pathways and incorporate these into holistic care plans, enabling personalized treatment approaches.

Finally, integrated therapeutic approaches could be explored, combining anti-inflammatory agents targeting microglia with antioxidants to mitigate oxidative stress activation or agents promoting remyelination and axonal repair.

By pursuing these directions, future research and therapeutic developments could significantly enhance the understanding and treatment of neurodegenerative disorders, improving outcomes for patients across multiple diseases.

## Data Availability

No new data were created or analyzed in this study. Data sharing is not applicable to this article.

## Supplementary Materials

The following supporting information can be downloaded at: https://www.mdpi.com/article/10.3390/ijms252312637/s1.
