# Supplementary material for: Should We Consider Neurodegeneration by Itself or in a Triangulation with Neuroinflammation and Demyelination? The Example of Multiple Sclerosis and Beyond
Source: Int J Mol Sci. 2024 Nov 25;25(23):12637. doi: 10.3390/ijms252312637 (PMC11641818; doi:10.3390/ijms252312637)
Supplement: Supplementary file 1 [file ijms-25-12637-s001.zip › ijms-3316365-supplementary.pdf]

	Multiple sclerosis	*Alzheimer's disease*<br>*Parkinson's disease*	*Metabolic syndrome*
Peculiar pathophysiology	Chronic active plaques, SELs<br>NAWM/NAGM<br>Cortical demyelination	Amyloid beta plaques/tau tangles (AD)<br>Alpha-synuclein (PD)	Leptin/Leptin resistance<br>Insulin resistance
Neuroinflammation	BBB disruption<br>Proinflammatory mediators<br>CD8+ cytotoxicity<br>Autoantibody-dependent cytotoxicity and opsonization<br>Meningeal inflammation<br>Overreactive microgliosis/astrogliosis<br>RNS/ROS/lactate production<br>Altered phagocytosis<br>Complement activation<br>Inflammasome activation	Chronic low-grade inflammation<br>BBB disruption<br>Reduced neurotoxin clearance<br>CD8+ cytotoxicity<br>Microglial priming, ROS<br>Reactive astrogliosis<br>Altered phagocytosis<br>Inflammasome activation<br>Iron overload	Chronic low-grade inflammation<br>BBB disruption<br>Microglial activation<br>↗ Proinflammatory leptin<br>↘ Anti-inflammatory adiponectin
Demyelination	Loss of BBB support<br>Loss of trophic support<br>Loss of protective barrier<br>Loss of electric insulator, nodal disruption<br>Myelin-associated growth inhibitory factors<br>Changes in lipid metabolism and species<br>Peroxisome deficiency<br>Iron overload and ferroptosis<br>Immune trigger (citrullination, myelin debris)<br>Immunocompetent OPC/OL	OPC block, OL disruption<br>Lipid dysregulation<br>Changes in lipid metabolism<br>Myelin defects<br>Microstructural white matter disorganization<br>Neuropathologic retrogenesis<br>Iron overload<br>Immunocompetent OL	OPC/OL block/loss<br>Myelin microstructural disruption<br>Hypomyelination
Neurodegeneration	Loss of axonally derived growth factors<br>Axonal non-receptivity to OPCs<br>Synaptic dysfunction	Axono-/Synaptopathy<br>Membrane destabilization/permeabilization and apoptosis due to protein misfolding<br>Neuronal hyperactivity<br>Excitoxicity (calcium overload)	Neuronal/ synaptic malfunctioning and atrophy due to impaired insulin and leptin signaling
Shared mechanisms	Oxidative stress, mitochondrial dysfunction<br>Ion imbalance (calcium)<br>Excitotoxicity (glutamate)<br>Metabolic reprogramming (ATP/lipids)<br>Energy deficit<br>"Don't eat me" signal<br>Virtual hypoxia, cell death<br>Cellular senescence	Oxidative stress<br>Mitochondrial dysfunction<br>Cellular senescence	Oxidative stress<br>Mitochondrial dysfunction

Table S1. Comparison of some pathophysiological mechanisms underlying the interplay between neuroinflammation, demyelination and neurodegeneration as well as shared mechanisms occurring in all cell types. The table also highlights peculiar features within the pathophysiology of each disease. AD = Alzheimer's disease, BBB = blood-brain barrier, NAWM = normal appearing white matter, NAGM = normal appearing gray matter, OPC = oligodendrocyte progenitor cell, OL = oligodendrocyte, PD = Parkinson's disease, RNS = reactive nitrogen species, ROS = reactive oxygen species, SELs = slowly expanding white matter lesions, ↗ = increased, ↘ = decreased.
